# JACUSA: site-specific identification of RNA editing events from replicate sequencing data

**DOI:** 10.1186/s12859-016-1432-8

**Published:** 2017-01-03

**Authors:** Michael Piechotta, Emanuel Wyler, Uwe Ohler, Markus Landthaler, Christoph Dieterich

**Affiliations:** 1 Max Planck Institute for Biology of Ageing, Joseph-Stelzmann Str. 9b, Cologne, 50931 Germany; 2 Berlin Institute for Medical Systems Biology, Max-Delbrück-Center for Molecular Medicine, Robert-Rössle-Strasse 10, Berlin, 13125 Germany; 3 Section of Bioinformatics and Systems Cardiology, Klaus Tschira Institute for Integrative Computational Cardiology at the Department of Internal Medicine III, University Hospital Heidelberg, Im Neuenheimer Feld 669, Heidelberg, 69120 Germany; 4 German Center for Cardiovascular Research (DZHK) - Partner site Heidelberg/Mannheim, Im Neuenheimer Feld 669, Heidelberg, 69120 Germany

**Keywords:** SNV, RNA editing, ADAR, APOBEC3, Variant calling

## Abstract

**Background:**

RNA editing is a co-transcriptional modification that increases the molecular diversity, alters secondary structure and protein coding sequences by changing the sequence of transcripts. The most common RNA editing modification is the single base substitution (*A*→*I*) that is catalyzed by the members of the Adenosine deaminases that act on RNA (ADAR) family. Typically, editing sites are identified as RNA-DNA-differences (RDDs) in a comparison of genome and transcriptome data from next-generation sequencing experiments. However, a method for robust detection of site-specific editing events from replicate RNA-seq data has not been published so far. Even more surprising, condition-specific editing events, which would show up as differences in RNA-RNA comparisons (RRDs) and depend on particular cellular states, are rarely discussed in the literature.

**Results:**

We present JACUSA, a versatile one-stop solution to detect single nucleotide variant positions from comparing RNA-DNA and/or RNA-RNA sequencing samples. The performance of JACUSA has been carefully evaluated and compared to other variant callers in an in silico benchmark. JACUSA outperforms other algorithms in terms of the F measure, which combines precision and recall, in all benchmark scenarios. This performance margin is highest for the RNA-RNA comparison scenario.

We further validated JACUSA’s performance by testing its ability to detect *A*→*I* events using sequencing data from a human cell culture experiment and publicly available RNA-seq data from *Drosophila melanogaster* heads. To this end, we performed whole genome and RNA sequencing of HEK-293 cells on samples with lowered activity of candidate RNA editing enzymes. JACUSA has a higher recall and comparable precision for detecting true editing sites in RDD comparisons of HEK-293 data. Intriguingly, JACUSA captures most *A*→*I* events from RRD comparisons of RNA sequencing data derived from Drosophila and HEK-293 data sets.

**Conclusion:**

Our software JACUSA detects single nucleotide variants by comparing data from next-generation sequencing experiments (RNA-DNA or RNA-RNA). In practice, JACUSA shows higher recall and comparable precision in detecting *A*→*I* sites from RNA-DNA comparisons, while showing higher precision and recall in RNA-RNA comparisons.

**Electronic supplementary material:**

The online version of this article (doi:10.1186/s12859-016-1432-8) contains supplementary material, which is available to authorized users.

## Background

RNA editing refers to co-transcriptional RNA base modifications that increase transcript sequence diversity without changing the underlying genome. Two types of single base modifications, namely adenosine to inosine conversions (*A*→*I*) and cytidin to uridine (*C*→*U*) conversions, have been characterised in detail over decades of research [1]. Both conversions are executed by two specific classes of RNA binding proteins (RBPs) that interact with their respective RNA targets: Adenosine deaminase acting on RNA (ADAR) catalyses *A*→*I* conversions, whereas APOBEC1 family members catalyse *C*→*U* conversions.

ADAR mediates the more frequent *A*→*I* editing by binding to double-stranded RNA and subsequent hydrolytic deamination of adenosine residues [1]. Most functional editing sites described so far are found in transcripts for neuronal transporters and channel proteins in the brain [2]. Herein, editing is critical for normal brain development and function. Specifically, ADAR-mediated editing of the GluA2 subunit of the mammalian AMPA receptor is an essential event [3]. Generally, inosine is interpreted as guanosine by the translation machinery, which may lead to codon substitutions in protein-coding sequences. Almost 100% of the human GluA2 transcripts are edited at codon position 607 which leads to a substitution of glutamine (CAG codon) with arginine (CIG codon) in the polypeptide chain. The introduction of a positive charge reduces calcium permeability in the mammalian AMPA receptor. In human, aberrant editing of the Q/R sites has been associated with death of motor neurons [4].

However, the vast majority of editing events takes places outside of coding regions [2]. Repetitive elements as well as 5’ and 3’ untranslated regions (UTRs) are the most frequent targets of RNA editing [5]. Especially Alu elements are targets of positionally unspecific abundant editing events [5, 6]. Alu repeats are short (≈ 300bp) mobile elements that are widespread in primates. Alu elements often co-occur in inverted pairs and form double-stranded RNA molecules after transcription, which constitute a favourable substrate for ADAR family members.

Taken together, site-specific RNA editing events may lead to amino acid substitutions by changing codons in coding sequences. Apart from its role in coding regions, RNA editing may also influence transcript splicing and structure and could have an effect on mRNA stability and nuclear export [2].

### Identification of RNA editing sites

The previously introduced RNA editing events are single nucleotide variants that can be detected from comparing genomic and transcriptomic sequencing data. RNA-DNA differences (RDDs) of the nucleotide frequency spectrum at a given location are the most direct way of identifying editing sites, whereas RNA-RNA comparison may pinpoint differential editing events across samples and conditions (RNA-RNA differences, in short RRDs). The availability of deep next-generation sequencing data enabled the transcriptome-wide discovery of RNA editing events. A direct comparison of gDNA and cDNA sequencing data has been proposed early on [7]. However, these early attempts suffered from the inherent artefacts of short read sequencing data and ambiguities in read mapping. For example, a re-analysis of the primary data of [7] revealed that close to 90% of the reported sites were false positives due to mapping and sequencing artifacts [8]. It was noted specifically that false editing calls were predominantly originating from base calls close to the start or end of reads, whereas true positives did not show this positional bias. Sequencing errors, read mapping errors and library preparation biases, which were introduced by ligation or amplification steps, all contribute to the high false positive rate. It is therefore essential to take these confounding factors into account or to remove them in a pre-processing step.

Several software solutions have been suggested for calling SNV sites: SAMtools/BCFtools [9], REDItools [10] and others (e.g. [11] and [12]). One particular common procedure for the identification of RNA editing is based on arbitrary thresholds for the number of minimal variant reads and minimal variant frequency (=10%) while at least a coverage of 10 reads is required [13].

Based on our previous experience from developing ACCUSA2 [14], we implemented a new software package, the **JA**VA framework for ac**cu**rate **S**NV **a**ssessment (JACUSA). JACUSA is a fast and precise solution for quantitative single nucleotide variant detection in RNA-DNA or RNA-RNA comparisons. JACUSA is primarily designed for the detection of position-specific editing events and readily integrates information from replicate experiments.

In the next sections, we will present our statistical framework and data processing steps in detail. We benchmark JACUSA on simulated data sets and compare its performance to other available and popular variant callers: REDItools [10], MuTect [15], and SAMtools/BCFtools [9]. We will then discuss the performance of JACUSA and the other tested variants callers in a controlled biological setting using sequencing data from ADAR knockdown experiments with human embryonic kidney (HEK-293) cells. Herein, several gDNA and cDNA libraries were sequenced to facilitate RNA-DNA and RNA-RNA comparisons based on Illumina sequencing data. Moreover, we made use of published RNA-seq data from *Drosophila melanogaster* fly brains that either originate from a wild type strain or a strain with a genetically ablated *dADAR* gene. With the Drosophila samples, we specifically look at the identifiability of editing events in protein coding exons in neuronal tissue where they have been reported previously [16].

## Implementation

In the following, we present the JACUSA software in detail, discuss the test statistics that supports replicate experiments and a set of positional filters that enable the pruning of false positive variants for a more accurate detection of SNVs. Equally important, we have implemented parallel and memory-efficient read processing routines for better performance and usability.

### Objective

**Fig. 1:**
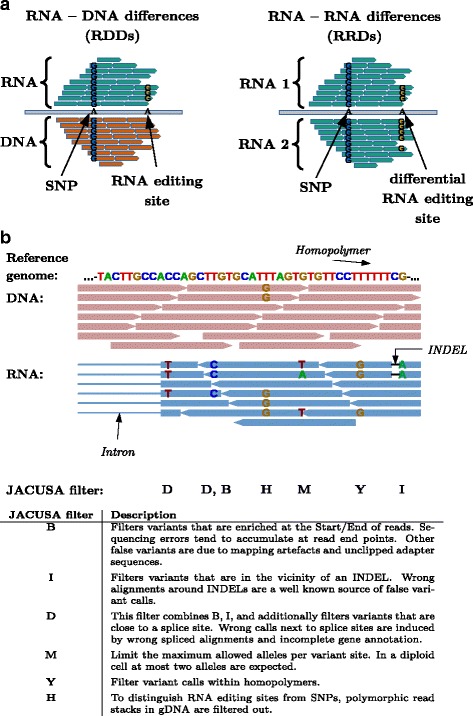
Possible nucleotide comparisons and implemented JACUSA filter. **a** Graphical representation of RNA-DNA differences (RDDs) and RNA-RNA-differences (RRDs) in head-to-head comparisons of sequencing data. **b** Possible sequencing artifacts and their respective JACUSA filters

The JACUSA software predicts single-nucleotide variant positions from head-to-head comparisons of read stacks/pileups from Illumina sequencing. In this manuscript, we focus on identifying nucleotide-level differences in RNA-DNA and RNA-RNA comparisons (see Fig. 1 a). Our method is robust to differences in read coverage, takes replicate information into account and avoids false calls by removing typical artifacts from short read data. We discuss the power of our approach specifically in the context of RNA editing.

### Statistical model

Previously, it has been shown that allele frequencies/counts are not accurately modeled by over-simplistic statistical models (e.g. a multinomial distribution) [17]. Typically, the observed variance will be higher than the theoretically expected variance in a multinomial model. This phenomenon is called overdispersion and will lead to false positive calls in variant detection. Therefore, we model DNA and RNA sequencing data with the Dirichlet-Multinomial distribution that accounts for overdispersion [18]. In the following, we use formulas and nomenclature defined in [19] adjusted to the alphabet of nucleotides *Σ*={*A*,*C*,*G*,*T*}. We define ***p***=(*p*_*A*_,*p*_*C*_,*p*_*G*_,*p*_*T*_) to be a random probability vector, such that *p*_*k*_:*k*∈*Σ* represents the base or allele probability for base *k* and the elements sum to 1. We can model ***p*** with a Dirichlet distribution $\mathcal {D}$ that has the parameter vector ***α***=(*α*_*A*_,*α*_*C*_,*α*_*G*_,*α*_*T*_):

1 $$\begin{array}{*{20}l} p(\boldsymbol{p}) &\sim \mathcal{D}(\alpha_{A}, \alpha_{C}, \alpha_{G}, \alpha_{T}) \end{array} $$

2 $$\begin{array}{*{20}l} &= \frac{\Gamma(\sum_{k} \alpha_{k})}{\prod_{k} \Gamma(\alpha_{k})} \prod_{k} p_{k}^{\alpha_{k} - 1} \end{array} $$

3 $$\begin{array}{*{20}l} \text{where } p_{k} &> 0 \end{array} $$

In [14], we estimated ***α*** from base calls and their respective base call quality score using an empirical Bayesian method. The Dirichlet distribution $\mathcal {D}$ is a conjugate of the multinomial distribution $\mathcal {M}$. Let ***x***=(*x*_*A*_,*x*_*C*_,*x*_*G*_,*x*_*T*_) represent the sum of base calls at some location and let ***x*** follow a multinomial distribution $\mathcal {M}(n, \boldsymbol {p}) = p(\boldsymbol {x}|\boldsymbol {p})$ where $n = \sum _{k} x_{k}$ is the total number of observed bases. By integrating over ***p*** we can combine ***x*** and ***p*** into the compound distribution that is called the Dirichlet-Multinomial:

4 $$\begin{aligned} DirMult(\boldsymbol{x}, \boldsymbol{\alpha}) := p(\boldsymbol{x}|\boldsymbol{\alpha}) &= \int p(\boldsymbol{x}|\boldsymbol{p}) p(\boldsymbol{p}|\boldsymbol{\alpha}) d\boldsymbol{p} \\ &= \frac{(n!) \Gamma(\alpha_{0})}{\Gamma(n + \alpha_{0})} \prod_{k \in \Sigma} \frac{\Gamma(x_{k} + \alpha_{k})}{\Gamma(x_{k} + 1) \Gamma(\alpha_{k})}, \end{aligned}  $$

where $\alpha _{0} = \sum _{k \in \Sigma } \alpha _{k}$.

An alternative interpretation of the Dirichlet-Multinomial is that of a hierarchical model:

$$\begin{array}{*{20}l} \boldsymbol{p} &\sim \mathcal{D}(\boldsymbol{\alpha}) \\ \boldsymbol{x} &\sim \mathcal{M}(\boldsymbol{p}) \end{array} $$

Let *D*={***x***_***1***_,***x***_***i***_,…,***x***_***N***_}:*i*∈{1,⋯,*N*} represent the base count vectors in *N* replicates and let ***x***_***i***_ be identically and independently distributed. Then ***α*** can be estimated from *D* by maximum likelihood estimation of $\mathcal {L}$:

$$ \mathcal{L}(\boldsymbol{\alpha}; D) = p(D|\boldsymbol{\alpha}) = \prod_{i} p(\boldsymbol{x_{i}}|\boldsymbol{\alpha}) $$

In order to model uncertainty of ***α*** we add a pseudocount term ***x***_***P***_ to the base call count vector: $\boldsymbol {\tilde {x}} = \boldsymbol {x} + \boldsymbol {x_{P}}$. The pseudocount term ***x***_***P***_ is calculated as a sum from observed quality score *q*_*BC*_ (i.e. variable terms) and a fixed noise term *ε* (=0.01) which models sequencing independent errors, which were derived empirically. *q*_*BC*_ is reported per base call as Phred quality score *q*_*BC*_, which is logarithmically related to the base-calling error probability *e*_*BC*_ [20]:

5 $$\begin{array}{*{20}l} e_{BC} &= Pr\{\text{wrong BC}\} \end{array} $$

6 $$\begin{array}{*{20}l} q_{BC} &= -10 \log_{10} e_{BC} \end{array} $$

7 $$\begin{array}{*{20}l} 1 - e_{BC} &= p_{BC} = Pr\{\text{called base}\} \end{array} $$

In JACUSA, we assume that the error probability *e*_*BC*_ is independent of the called base. That is why, the error probability of an uncalled base is given by:

8 $$ \frac{e_{BC}}{3} = Pr\{\text{uncalled base}\}  $$

Using these considerations and referring to a specific base call by *l*, we define ***x***_***P***_ as:

9 $$ \boldsymbol{x_{P}} = \sum_{1 \geq l \geq n} \left\{ \begin{array}{ll} \epsilon + \frac{e^{l}_{BC}}{3} & \text{for each uncalled base} \\ 0 & \text{otherwise.} \end{array}\right.  $$

#### Statistical test

We define our test statistic as a likelihood ratio of two samples *j*∈{*I*,*I* *I*} where the data of each sample is defined as the pseudocount adjusted base call vectors $\tilde {D}^{j} = \left \{\boldsymbol {\tilde {x}^{j}_{1}}, \boldsymbol {\tilde {x}^{j}_{i}}, \ldots, \boldsymbol {\tilde {x}^{j}_{N_{j}}}\right \}$. We use the Dirichlet-Multinomial distribution to model $\tilde {D^{j}}$ and estimate ***α*** ^***j***^ as explained in the previous section. We test against the null hypothesis *H*_0_ that both samples originate from the same underlying distribution. The log-likelihood score function *z* (Eq. 10) will have higher values, the better each of the parameter vectors ***α*** ^***I***^ and ***α*** ^***II***^ represent the underlying data implying that each sample *I* and *II* has a different underlying distribution.

10 $$ z = \log \frac{DirMult(\boldsymbol{\alpha^{I}}; \tilde{D}^{I}) \cdot DirMult(\boldsymbol{\alpha^{II}}; \tilde{D}^{II})}{DirMult(\boldsymbol{\alpha^{I, II}}; \tilde{D}^{I}) \cdot DirMult(\boldsymbol{\alpha^{I, II}}; \tilde{D}^{II})}  $$

The coverage between two pileups may differ extremely between RNA-seq samples. This will sometimes lead to an overestimation of confidence in the base call vector $\boldsymbol {\tilde {x}}$ for the sample with higher coverage. We mitigate this phenomenon by adjusting the underlying read stacks. In essence, large coverage differences between a single nucleotide count vector $\tilde {D}_{homo}$ and count vectors with two or more nucleotides $\tilde {D}_{hetero}$ are evened out by replacing the original $\tilde {D}_{homo}$ with a copy of $\tilde {D}_{hetero}$ where all variant positions have been replaced by the reference nucleotide. Depending on the encountered read stacks, JACUSA automatically switches to the optimal comparison mode.

#### Implemented filters

Many false positive RDD calls in RNA editing studies are related to mapping artefacts [8]. Short read mappers tend to produce incorrect alignments around INDEL positions that may be falsely identified as variant sites. Tools such as GATK [21] allow to adjust for this effect by sensitive local realignment of reads that contain INDELs. Other false variant calls originate from uneven base call error distributions along short reads. This may be related to sequencing technology where base calls at read ends are less reliable. In JACUSA, we have implemented a panel of simple threshold based filters to remove the aforementioned and other artefacts (see Fig. 1). Our filters (D,B,I,Y) monitor the distance *d* of a given candidate site to relevant read features such as start/end, INDEL positions, homopolymeric regions, and splice sites and remove the candidate site from further consideration if a proportion *r* of all reads falls below the given distance cutoff ≤*d*.

Generally, it is common practice to define RDDs for homozygous genomic positions (filter H) and with less than three distinct base types (filter M). Moreover, we strongly recommend to remove PCR-duplicate reads from the input read sets to minimize biases, which are introduced by PCR amplification biases, before the actual JACUSA run (see Additional file 1: Section 4.4).

### In silico benchmark

**Fig. 2:**
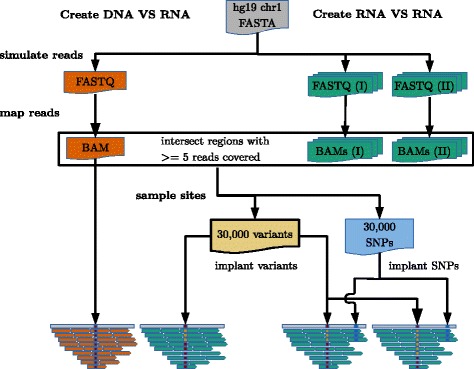
Summary of data generation for in silico benchmark. Description of the data generation for in silico RDD and RRD detection. In total 1 DNA sample and 2x15 RNA samples (I+II) are simulated. DNA and RNA reads are simulated from chromosome 1 of human genome. Candidate SNPs and variant sites are created in regions that are covered by all simulated BAM files. Depending on the comparison, SNPs and variant sites are inserted into BAM files. SNPs are only implanted in the cDNA vs. cDNA setup and function there as noise

We define two benchmark scenarios (Fig. 2): 1) gDNA vs. cDNA simulates data for the identification of RNA-DNA differences (RDDs) and 2) cDNA vs. cDNA generates data for the identification of RNA-RNA differences (RRDs). The gDNA vs. cDNA represents the typical setup for the detection of RNA editing sites. In this scenario, editing sites have been only implanted into the cDNA BAM file(s). In the cDNA vs. cDNA data setup, both data sources may contain base substitutions at different frequencies. This scenario can be interpreted as allele-specific expression or dynamic RNA editing changes. Herein, variants with pairwise different base frequencies (*Δ*>0.1) have been implanted into each corresponding cDNA BAM file. Additionally, to make the identification of variants more challenging, SNPs with pairwise similar base frequencies have been included into each cDNA BAM file (see Fig. 2). We use the human reference genome (hg19, chromosome 1) as a template to simulate genomic DNA (gDNA) and RNA-Seq reads. In total, 60,000 non-overlapping sites have been randomly chosen based on sufficient read coverage 5≥*c*≥1000 and read mapping quality ≥20 in all simulated BAM files. The initial candidate set of non-overlapping sites has been divided into 30,000 variant and SNP sites, respectively. Each site is modeled with a variant target frequency as shown in Tables 1 and 2.

**Table 1:** Detailed statistics of the implanted sites for RNA-DNA-difference (RDD) benchmark setup

	gDNA	cDNA
Variants	0	30,000 positions
*Δ* variant freq.		≥0.01

**Table 2:** Detailed statistics of the implanted sites for RNA-RNA-difference (RRD) benchmark setup

	cDNA (I)	cDNA (II)
SNPs	30,000 positions	
*Δ* variant freq.	≈0	
Variants	30,000 positions	
*Δ* variant freq.	≥0.1	

Moreover, we introduce additional variability by sampling the target variant frequencies from a Beta-Distribution with concentration parameter *β*∈{10,50,100} representing sites with high, medium, and low variability around the expected target frequencies. Another parameter of our benchmark is the number of RNA-seq replicates. We benchmarked all scenarios with cDNA samples from 1 to 5 replicates. Each replicate setup is simulated 3 times, which amounts to 15 RNA-seq FASTQ files per benchmark (see Table 3 for details).

**Table 3:** Description of in silico samples

	gDNA	cDNA (I)	cDNA (II)
Library type	gDNA, paired-end	RNA-Seq, paired-end	
Read length	2x100nt	2x100nt	
Read count/coverage	30x	15,000,000 raw reads	
# of FASTQ files	1	3x5	

Additional details on the benchmark setup are given in Additional file 1: Section 3 and Table 4.

**Table 4:** Summary of variant callers used for available benchmarks and their support for replicates

Variant caller	Support for	gDNA vs. cDNA	cDNA vs. cDNA
	replicates		
SAMtools/BCFtools	x	x	x
REDItools		x^a^	
MuTect		x	
JACUSA	x	x	x

^a^The REDItools package supplies multiple methods to identify RNAediting sites. We employed the REDItoolDenovo.py script because it provides a test-statistic. This method only utilizes RNA-Seq reads

### Sequencing the HEK-293 genome and transcriptome

#### HEK-293 genome sequencing

Genomic DNA was isolated from Flp-In T-REx HEK-293 cells (Invitrogen) using the GenElute Mammalian Genomic DNA Miniprep Kit (Sigma-Aldrich). DNA was fragmented in the BioRuptor Plus (Diagenode, setting “high” in a total volume of 150*μ* *l* (concentration 25*n* *g*/*μ* *l*), with 24 cycles (30 seconds on, 30 seconds off) in a 4 °C water bath, including a brief centrifugation after 12 cycles. The resulting fragmented DNA was converted to a sequencing library using the TruSeq DNA kit (Illumina) with PCR enrichment and sequenced on a Illumina HiSeq 2500 machine. In total >10^9^ reads have been sequenced (see Additional file 1: Table S3). The gDNA-seq data have been deposited in the NCBI SRA under accession SRP050149.

#### HEK-293 transcriptome sequencing

HEK-293 strand-specific RNA-Seq data from [22] has been downloaded and processed as explained in Fig. 5. We used the hg19 human genome and ENSEMBL 75 annotation for mapping. The TopHat2 [23] mismatch parameter was set to 10 and reads with more than 5 mismatches were filtered subsequently (see Additional file 1: Section 4.3). Alu regions have been download and extracted from RepeatMasker annotation Ver. 4.0.2. [24].

### RNA-seq data from Drosophila fly heads

We obtained published replicate paired-end RNA-seq data from Drosophila fly heads [25] (2 x 100nt, unstranded, accessions codes: NCBI SRA SRR485862-5). The *Drosophila melanogaster* genome carries only a single copy *dADAR* gene. Two replicate RNA samples were generated from flies with wildtype and null alleles of *dADAR* (2 replicates each, FM7a strain background). We processed the data in the same way as the HEK-293 data sets using the Ensembl 75 Drosophila genome and annotations.

## Results

### In silico benchmark

We use two benchmark scenarios (Fig. 2) to compare JACUSA with other popular variant callers: REDItools, SAMtools/BCFtools, and MuTect. The gDNA vs. cDNA scenario works with all variant callers while the cDNA vs. cDNA comparison scenario could be only tested with SAMtools/BCFtools and JACUSA. Equally important, SAMtools/BCFtools and JACUSA are the only two variant callers that support replicates in our benchmark. More details on the benchmark setup and how others and our software were used are given in section 3.1 of the Additional file 1.

#### Detection of SNVs in RNA-DNA comparisons

**Fig. 3:**
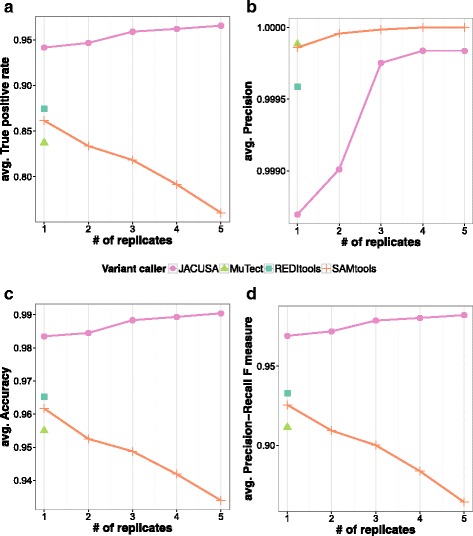
Benchmark results for in silico RDD detection. **a** True positive rate, **b** Precision, **c** Accuracy plot, and **d** F-measure

When no replicates are used, JACUSA shows a 6−10*%* higher true positive rate (TPR) as compared to the other tested methods while being competitive at the level of precision (see Fig. 3 a, b). The single replicate scenario is highly relevant in practice, as RNA-seq replicate counts are typically low in RDD studies in the clinics. We specifically used the accuracy measure and the F-score to evaluate the balance between precision and true positive rate (see Additional file 1: Section 3.2). The main difference between these performance measures is that the accuracy measure includes the number of true negatives.

Of all tested methods, JACUSA scores the highest in terms of accuracy and F-score (see Fig. 3). The tradeoff between TPs and FPs can be nicely observed for the comparison of MuTect and REDItools. While REDItools shows a higher TPR (87,45*%* compared to 83,73*%* of MuTect, Fig. 3 a), the precision is slightly higher for MuTect (99,99*%* compared to 99.96*%* forREDItools, Fig. 3 b). SAMtools/BCFtools scores third in terms of TPR and achieves together with MuTect the highest precision of 99,99*%*.

JACUSA takes advantage of replicate information and shows a steady increase in performance with the number of employed replicates. SAMtools/BCFtools on the other hand displays only growing precision with increasing number of replicates and the remaining performance measures are decreasing. The drop in performance is highest for 5 replicates and amounts to more than 15% of TRP. JACUSA consistently performs better than SAMtools/BCFtools in terms of TPR, F-score, and accuracy (see Fig. 3 a-d).

Additional results and details are given in Additional file 1: Section 3.3.

#### Detection of SNVs in RNA-RNA comparisons

In the cDNA vs. cDNA scenario we replace the single gDNA sample by one or more cDNA samples with variant sites where the target frequency differs by more than 10% between both cDNA pools. We introduce polymorphic positions of equal target frequency into both samples. The goal of this benchmark is to test the ability of the respective variant caller to distinguish between variant sites with a target frequency difference of *Δ*>0.1 and polymorphic positions with equal target frequency.

**Fig. 4:**
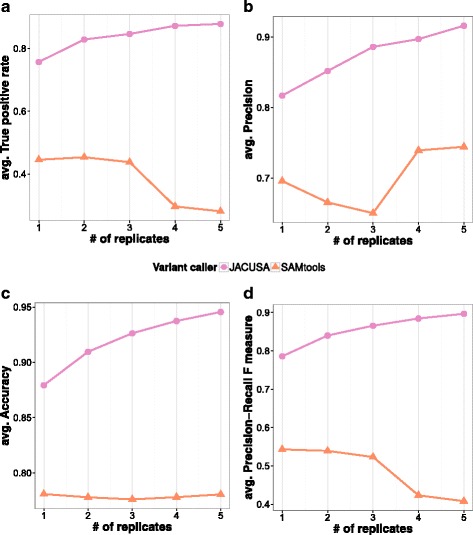
Benchmark results for in silico RRD detection. **a** True positive rate, **b** Precision, **c** Accuracy plot, and **d** F-measure

As before, we evaluate the variant callers by comparing overall performance measures such as F-score and accuracy. A general observation is the lower accuracy in Fig. 4 a for calling variant sites in cDNA vs cDNA comparisons. In essence, it is a much harder task than contrasting gDNA vs. cDNA samples. In terms of true positive rate, JACUSA outperforms SAMtools/BCFtools in this scenario by at least 40% when replicates are available and by over 35% when no replicates are available (see Fig. 4 a).

**Fig. 5:**
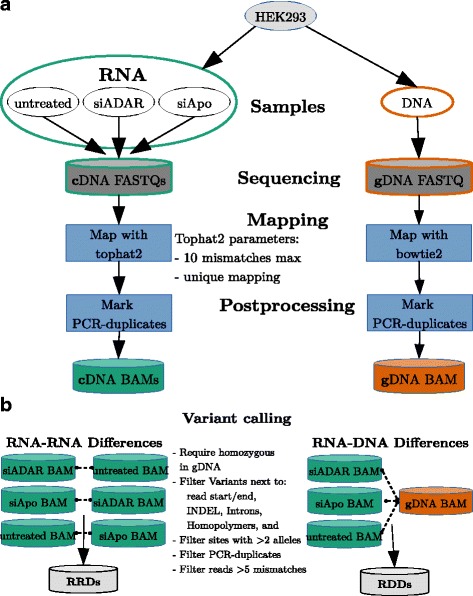
Analysis workflow to identify RNA editing sites in matched gDNA and cDNA HEK-293 samples. **a** Sequenced reads are mapped and PCR-duplicates are removed. **b** Single nucleotide variants are called for all sensible combinations of gDNA vs. cDNA and cDNA vs. cDNA BAM files

Next, we combined true positives (TPs) and false positives (FPs) into composite measures and observed 10−16*%* better average accuracy for JACUSA (see Fig. 4 c). This is even more pronounced for the F-score measure, where JACUSA is performing at least 20% better in all tested replicate scenarios (see Fig. 4 d).

Additional results and details are given in Additional file 1: Section 3.4. A general overview on the single thread runtime of each tested software is shown in Additional file 1: Section 3.6.

### Editing in HEK-293 cells

To assess the performance of JACUSA in practice, we designed a controlled experiment to generate sequencing input data from cell culture experiments (see Fig. 5 a). Briefly, we resequenced the genome of HEK-293 cells to an average coverage of 30x (gDNA data). We obtained matching cDNA data from our previously published study [22]. Cells were either untreated or have been subjected to siRNA knockdown experiments targeting either ADAR 1+2 (siADAR) or APOBEC3 B,C, and F (siAPOBEC3). The ADAR and APOBEC3 family members have been previously observed as mRNA-binding proteins in a transcriptome-wide proteomics screen of the same cell type [26]. However, the APOBEC3 familiy members did not show significant C-to-U RNA editing activity in our assays.

Subsequently, we conducted gDNA vs. cDNA comparisons on the aforementioned data sets and predicted RNA editing sites with SAMtools/BCFtools, MuTect, and JACUSA. For each variant caller, we selected optimal thresholds for the HEK-293 data set based on our results from the in silico data set: gDNA vs. cDNA score threshold is 1.15 and cDNA vs. cDNA threshold is 1.56. Additional details on selecting score thresholds are given in Additional file 1: Section 3.5.

For MuTect and REDItools we adopted a strategy presented in [12] to utilize replicate information by first calling variants on pooled biological replicates and finally filtering and requiring that the primarily identified variants are present in all replicates. We used JACUSA as explained in Fig. 5 b to detect RNA editing sites utilizing replicates. Additional details on the workflow and results are given in Additional file 1: Section 4 and following. All editing site predictions are listed in Additional file 2.

#### Calling RDDs from HEK-293 data

**Fig. 6:**
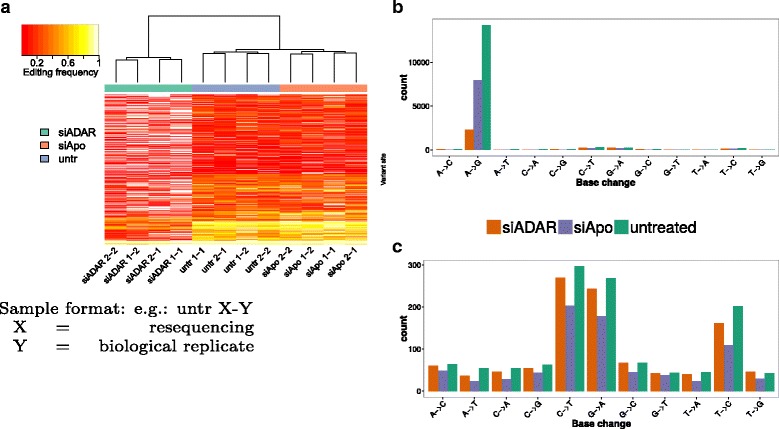
Results for RDD predictions in HEK-293 cells. **a** Comparison of editing frequency between biological and technical replicates of all samples. **b** Detected SNVs stratified by base changes in the HEK-293 RDD comparison. **c** Same as **b** but dominating *A*→*G* base transitions have been removed to focus on other lower-level base changes

In total, 2 biological replicates have been created per condition and were sequenced twice to assess the biological and technical variability. By computing RDDs on each replicate with JACUSA, we could show an excellent agreement among replicates from the same condition (see Fig. 6 a). Subsequently, we merged all technical replicates and identified our definite list of RDDs from comparing one gDNA vs. two biological replicate samples for each condition.

Our comparison of gDNA vs. RNA from untreated cells yielded 15,461 variant sites for JACUSA (with a proportion of 92.2*%* *A*→*G* sites, see Fig. 6 b and Table 5). This number drops to 8722 for the siAPOBEC3 RNA samples (91.2% *A*→*G*) and, as expected, to 3371 sites for the siADAR RNA samples.

**Table 5:** Predicted RDDs for each treatment of HEK-293 cells. Fraction of *A*→*G* RNA editing sites is provided in parenthesis

	gDNA vs. treatment		
Variant caller	untreated	siADAR	siAPOBEC3
SAMtools/BCFtools	11,191 (93.3%)	2117 (68.9%)	6423 (92%)
MuTect	7605 (92.5%)	1793 (69.4%)	4181 (90.2%)
REDItools	11,900 (90.1%)	2729 (59.7%)	6985 (88.6%)
JACUSA	15,461 (92.2%)	3371 (68.3%)	8722 (91.2%)

The siAPOBEC3 transfection experiment (mock) already leads to a reduction of editing sites. Editing levels are further reduced by targeting the correct enzyme class (siADAR experiment).

Interestingly, the non *A*→*G* sites identified by JACUSA (1203 in total, Fig. 6 c) consist mainly of three base substitutions: *C*→*T* (24.7*%*) editing is a known but rare modification that is mediated by APOBEC1 [27] and *T*→*C* and *G*→*A* variants (39.2%), which are the reverse complement versions of the canonical editing events.

JACUSA identified the highest number of RDDs (15,461 vs 11,191 for SAMtools/BCFtools) and showed a comparable fraction of *A*→*G* sites (92.2% vs 93.3% for SAMtools/BCFtools) among all tested variant callers (see Table 5).

MuTect identified far fewer RDDs (≈25*%* less) in comparison to the other variant callers while achieving second highest fraction of *A*→*G* sites (92.5*%*). This is in line with the in silico benchmark results on MuTect indicating a high precision but a lower recall. In summary, all variant callers identify RDDs with a fraction of *A*→*G* sites in the range of 90.1 and 93.3%, while the total number of variants varies greatly from 7605 (MuTect) up to 15,461 (JACUSA).

#### Agreement between RDD calls

**Fig. 7:**
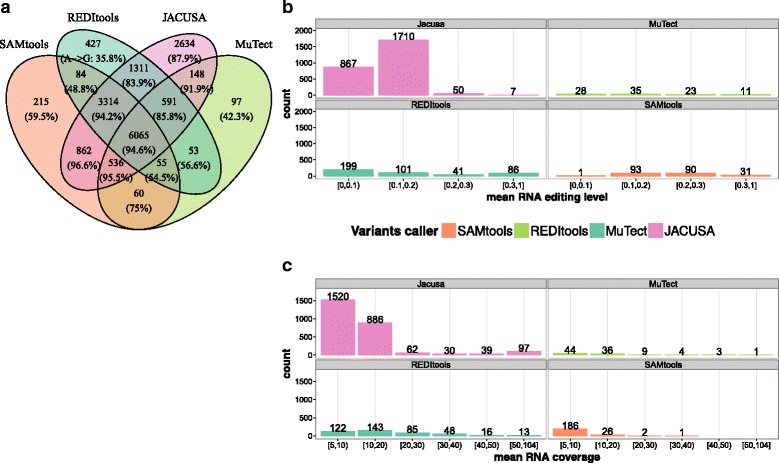
Comparison of variant callers in HEK-293 cells. **a** Overlap of RDD variants identified on HEK-293 untreated cells by all tested variant callers. The fraction of *A*→*G* is given in the parenthesis in each segment. **b** Distribution of average editing level of *A*→*G* sites that are exclusively identified by tested variant caller. **c** Distribution of average RNA coverage at exclusively predicted *A*→*G* sites

All four software solutions report a set of 6064 shared RDD sites for the untreated RNA sample, which show a high proportion of *A*→*G* sites (94.6*%*) (see Fig. 7 a). The second largest overlap of 3314 RDD predictions is seen for SAMtools/BCFtools, REDItools, and JACUSA (94.2*%* *A*→*G* sites). Strikingly, JACUSA identifies 2,634 additional RDDs, which are not reported by any other software tool and yet attain a proportion of 87.9*%* *A*→*G* sites. This is far more than for sites that were exclusively reported by SAMtools/BCFtools (59.5*%* *A*→*G* sites), REDItools (35.8*%*) or MuTect (42.3*%*).

Moreover, a detailed assessment of RDDs sites, which have been exclusively reported by JACUSA, shows a low mean editing level and mean coverage (see Fig. 7 b and c).

#### Response of RDD calls in ADAR knockdown

To control the effect of any siRNA knockdown treatment on RNA editing levels (see Fig. 6 b), we contrast editing levels of RDDs between siAPOBEC3 and siADAR samples. As mentioned earlier, JACUSA had identified 8722 RDDs in cells treated with siAPOBEC3 (siApo) of which 7953 were *A*→*G* substitutions. We classify these *A*→*G* sites as true positives if they show a drop in their editing frequency in an siADAR vs. siAPOBEC3 knockdown.

**Fig. 8:**
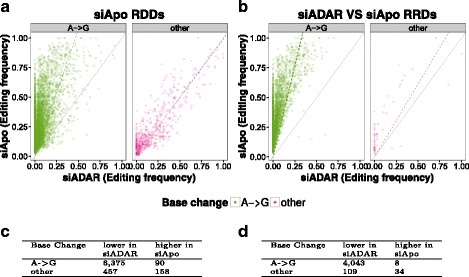
Properties of RDDs in HEK-293 cells. **a** Comparison of editing frequency of siADAR samples and RDDs detected in siAPOBEC3 (siApo) treated cells. (*Dashed line*(s) correspond(s) to regression line(s)) **b** Editing frequency of sites that are identified as divergent in RRD comparison of treatments. Tables **c** + **d** show details of editing frequencies statistics for scatterplots **a** and **d**, respectively

As shown in Table 6, we could assess editing level changes on 7084 RDD sites that had sufficient read coverage in both siRNA knockdown data sets (5 reads per position per replicate in siAPOBEC3 and siADAR samples). JACUSA identifies the highest number of RNA editing sites (6,466) out of which ≈98*%* show lower editing levels in siADAR samples than in samples from siAPOBEC3 treated cells. This means that JACUSA reports 6375 true positive *A*→*G* sites out of a set of 6,466 predicted sites, the highest among all tested variant callers. Figure 8 a and c depict this important result for each individual site. The clear shift of editing frequency was specific to *A*→*G* and could not be observed for any other base substitution (see Table c in Fig. 8). In summary, JACUSA identifies at least >20*%* more editing sites than any tested variant caller while its editing sites show an equal responsiveness to ADAR knockdown treatment.

**Table 6:** Comparison of average editing levels of detected RDDs on siADAR and siAPOBEC3 (siApo) treated HEK-293 cells

	RDDs in	Covered in	*A*→*G*	Avg. Editing level
Variant caller	gDNA vs. siApo	siADAR vs. siApo	Editing Sites	siADAR < siApo
SAMtools/BCFtools	6423	5066 (78.87%)	4691 (92.60%)	4630 (98.700%)
MuTect	4181	3415 (81.68%)	3087 (90.40%)	3043 (98.575%)
REDItools	6985	5823 (83.36%)	5180 (88.96%)	5099 (98.436%)
JACUSA	8722	7084 (81.22%)	6466 (91.28%)	6375 (98.593%)

#### Detection of differential RNA editing from RNA-RNA comparisons

**Fig. 9:**
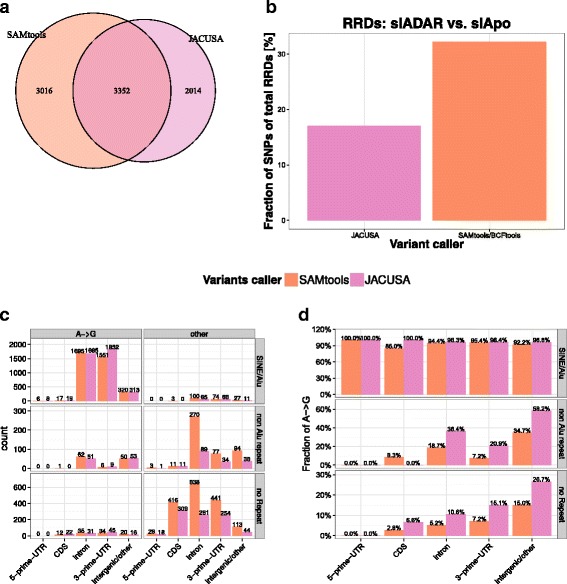
Properties of RRDs in HEK-293 cells. **a** Overlap of RRDs identified on siADAR and siAPOBEC3 treated HEK-293 cells. **b** Fraction of RRDs that are non homozygous in gDNA. **c** Gene location and repeat annotation of identified variants. **d** Fraction of *A*→*G* sites among identified variants

Another JACUSA application is to detect sites of differential RNA editing from RNA-seq data only. This could be effected through a direct assessment of RNA-RNA differences (RRD) in the absence of genomic sequencing data. We reasoned that one way to validate RRD site detection and ultimately differential *A*→*G* editing is to use our available RNA/DNA-seq data in the following way:

We screen our samples from siADAR and siAPOBEC3 knockdowns for RRDs. Our assumption is that APOBEC3 family members do not influence *A*→*G* editing and siRNA transfection effects cancel out in this comparison. “True” *A*→*G* editing sites should show a lower editing frequency in the siADAR knockdown. For the siADAR vs. siAPOBEC3 comparison, SAMtools/BCFtools predicts 6368 RRD sites and JACUSA predicts 5366 RRD sites (see Table 7). Out of these, 3352 RRDs are predicted by both SAMtools/BCFtools (52.6*%* of all SAMtools/BCFtools predictions) and JACUSA (64.5*%* of all JACUSA predictions) (see Fig. 9 a).

**Table 7:** Summary of all detected RRDs for all possible treatment combinations on HEK-293 cells

Variant caller	siADAR vs.	siADAR vs.	siApo vs.
	siApo	untreated	untreated
SAMtools/BCFtools	6368	8195	7462
JACUSA	5366	6977	2701

Subsequently, we retained RRDs that had at least 10x read coverage in the gDNA sample and checked if predicted sites are homozygous in the genome.

Sites that are not homozygous in DNA represent putative SNPs and are typically removed from the candidate set when identifying RNA editing sites in RDD comparisons. As this information is not visible to SAMtools/BCFtools and JACUSA, we reasoned that a lower fraction of SNP sites among identified RRDs would indicate a better performance on calling differential RNA editing events. In essence, JACUSA precision is at 83.0% (4284 true sites vs 5161 candidate sites) Table 8 while SAMtools/BCFtools attains only 67.8% (4088 true sites vs. 6026 candidate sites).

**Table 8:** Comparison of average editing levels of RNA editing sites that have been identified as RRDs in siADAR and siAPOBEC3 (siApo) treated HEK-293 cells

Variant	RRDs in	Covered	Homozygous	*A*→*G*	Avg. Editing level
caller	siADAR vs. siApo	in gDNA	in gDNA	Editing Sites	siADAR < siApo
SAMtools	6368	6026 (94.6%)	4088 (67.8%)	3838 (93.9%)	3731 (97.2%)
JACUSA	5366	5161 (96.2%)	4284 (83.0%)	4051 (94.6%)	4043 (99.8%)

We compared the fraction of RRDs that after coverage filtering were potential SNPs and found that SAMtools/BCFtools predictions contained 15% more putative polymorphic sites than JACUSA (see Fig. 9 b).

In summary, RRDs predicted by JACUSA showed a lower overlap with potential polymorphic sites and the fraction of *A*→*G* editing sites was higher than the candidates called by SAMtools/BCFtools. The editing frequency of 4,043 *A*→*G* sites was smaller in siADAR treated cells whereas only 8 would show a higher editing frequency in siADAR treated cells (see Fig. 8 b). The clear shift of editing frequency was specific to *A*→*G* and could not be observed for any other base substitution (see Tables in Fig. 8 b+d).

#### Editing events across genomic features

Another important aspect is the genomic distribution of our editing predictions. We stratified our RDD and RRD predictions by gene-centric (exon, introns, etc.) and repeat-centric categories (Alu, non-Alu and no repeat regions). As expected, most RDD predictions are made in regions that are annotated as Alu repeats. Prediction accuracy drops dramatically for non-Alu repeat regions and even more so for non-repeat regions. For details see Additional file 1: Tables S5-S8. This holds true for all four tested SNV callers. This effect seems to be independent of gene-centric features and strongly correlates with repeat type. We observed that most RDD sites in non-repeat regions cannot be explained by *A*→*G* editing. We also cannot exclude the possibility that HEK-293 cells generally show very little RNA editing in non-Alu regions. Nevertheless, JACUSA identifies most *A*→*G* sites in absolute numbers.

The same phenomenon becomes more evident for the RRD comparisons (see Additional file 1: Tables S9 and S10). Herein, hardly any *A*→*G* sites are predicted in non repeat regions, by both SAMtools and JACUSA.

#### Differential editing in Drosophila fly heads

**Fig. 10:**
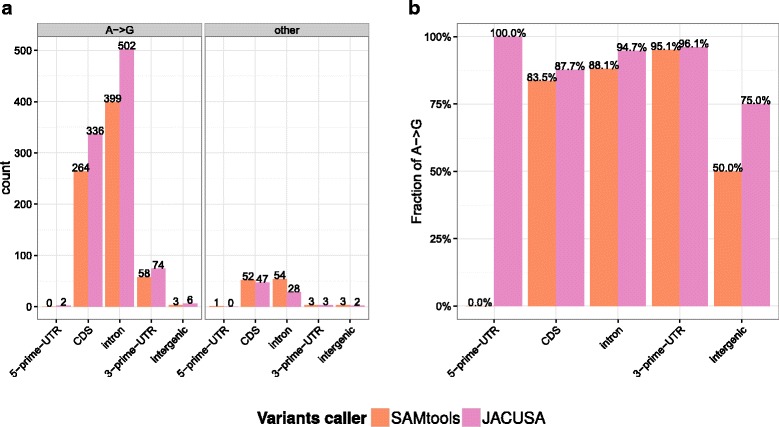
Properties of RRDs identified in *Drosophila melanogaster* samples by comparing dADAR-/- and wildtype strains. **a** Gene location and repeat annotation of RRDs. **b** Fraction of *A*→*G* sites among identified RRDs

We reasoned that our HEK-293 cell data sets could be complemented by an independent data set with a controlled experimental design for testing RRD site discovery. To this end, we analysed published RNA-seq data from Drosophila fly heads [25]. Rodriguez et al. use a genetic approach to ablate the activity of the single copy *dADAR* gene in the fruit fly (human *ADARB1* homolog). This is a favorable system for fine-mapping editing sites as editing activity depends only on a single enzyme in the fruit fly. Moreover, editing in coding exons, which are expressed in the fly brain, has been described previously [16]. In summary, JACUSA detected 931 RRD candidate sites (see Additional file 1: Tables S11 and S12) while SAMtools/BCFtools predicted 781 RRD candidates. However, while the vast majority (92.1%) of JACUSA RRD sites are *A*→*G* sites, just 86.3% of all SAMtools/BCFtools predictions are (see Fig. 10). Overall, JACUSA predicted 383 RDD sites in coding exons. A closer inspection showed that 336 (87.7%) of these are bona fide *A*→*G* sites (see Additional file 1: Tables S11 and S12). This analysis demonstrated that JACUSA is able to accurately predict editing events in RNA-RNA comparisons on an independent data set as well. All editing site predictions are listed in Additional file 3.

## Conclusion

In this manuscript, we have presented JACUSA as an accurate and fast one-stop solution to identify site-specific SNV events in matched sequencing samples. JACUSA outperformed other SNV callers in an in silico benchmark that assessed SNV calling performance in terms of identifying site-specific RNA-DNA differences (RDDs) and RNA-RNA differences (RRDs). While the first benchmark is the typical scenario for identifying RNA editing sites from homozygous genomic positions, the second benchmark represents another interesting case of identifying condition specific changes in editing frequencies.

JACUSA shows the best recall and competitive precision in comparison to all tested software solutions. The performance gain over its competitors is especially visible for the detection of RNA-RNA differences. In terms of recall, JACUSA outperforms SAMtools/BCFtools in the RRD scenario by at least 40% when replicates are available and by over 35% when no replicates are available. Intriguingly, this is not at the expense of precision which is at least 10% better over all tested number of replicates.

In practice, we tested JACUSA in a controlled experimental setup where we generated DNA and RNA-seq data from HEK-293 cells. Similar to the in silico benchmark, we first identified candidate sites of RNA editing via RDD comparisons and checked if their editing frequency would respond to changes in ADAR protein levels by siRNA knockdown experiments. With this setup, we could nicely demonstrate that JACUSA has a better recall and comparable precision to other tested variant callers in identifying *A*→*G* editing sites in RNA-DNA comparisons.

Subsequently, we assessed the RRD or differential editing scenario by predicting SNVs between replicate siAPOBEC3 and siADAR RNA samples. Again, JACUSA overall predicts more sites in homozygous DNA positions and a greater proportion of *A*→*G* editing sites than SAMtools (83.0% vs 67.8%) in this RNA-RNA comparison scenario on HEK-293 RNA-seq data.

These results were further corroborated by looking at an independent RNA-seq data set from *Drosophila melanogaster* heads. Herein, JACUSA reports the highest number of RNA editing sites (857 vs 674) with much higher precision (92.1% vs 86.3% of all RRD sites).

In summary, JACUSA is a versatile software for the precise and sensitive detection of single nucleotide level differences in DNA-RNA as well as RNA-RNA comparisons from Illumina sequencing data. In this manuscript, we have specifically explored its excellent ability to detect site-specific RNA editing events.

## Availability and requirements

gDNA-seq data have been deposited in the NCBI SRA under accession SRP050149.

- **Project name:** JACUSA
- **Project home page:** https://github.com/dieterich-lab/JACUSA
- **Operating system(s):** N/A
- **Programming language:** JAVA 1.6
- **Other requirements:** none
- **License:** GPL-3.0

## Additional files

Additional file 1 Supplementary Text. (PDF 2816 kb)

Additional file 2 Excel Spreadsheet with predictions on HEK-293 DNA and RNA sequencing data. (XLS 8867 kb)

Additional file 3 Excel Spreadsheet with predictions on Fly head RNA sequencing data. (XLS 259 kb)

## Abbreviations

ADAR: Adenosine deaminases that act on RNA
RDD: RNA-DNA-differences
RRD: RNA-RNA-differences
RBP: RNA binding protein
SNP: Single nucleotide polymorphism
SNV: Single nucleotide variant
