# Supplementary material for: JACUSA: site-specific identification of RNA editing events from replicate sequencing data
Source: BMC Bioinformatics. 2017 Jan 3;18:7. doi: 10.1186/s12859-016-1432-8 (PMC5210316; doi:10.1186/s12859-016-1432-8)
Supplement: Additional file 1 — Supplementary Text. (PDF 2816 kb) [file 12859_2016_1432_MOESM1_ESM.pdf]

Supplementary Material

Piechotta Michael
Wyler Emanuel
Ohler Uwe
Landthaler Markus
Dieterich Christoph

November 17, 2016

1 Acronyms

cDNA complementary DNA

gDNA genomic DNA

RDD RNA-DNA difference

RRD RNA-RNA difference

SNP Single nucleotide polymorphism

SNV Single nucleotide variant

2 JACUSA internals

2.1 Estimating parameters of the Dirichlet-Multinomial

Let $D = \{\boldsymbol{x_1}, \boldsymbol{x_i}, \ldots, \boldsymbol{x_N}\} : i \in \{1, \cdots, N\}$ represent the base count vectors in N replicates and let $\boldsymbol{x_i}$ be identically and independently distributed then $\boldsymbol{\alpha}$ can be estimated from D by maximum likelihood estimation of $\mathcal{L}$:

$$\mathcal{L}(\boldsymbol{\alpha}; D) = p(D|\boldsymbol{\alpha}) = \prod_i p(\boldsymbol{x_i}|\boldsymbol{\alpha})$$

We estimate $\boldsymbol{\alpha}$ by the Newton-Raphson method ([1]) that optimizes the log-likelihood function $\log p(D|\boldsymbol{\alpha})$:

$$\begin{aligned} n_i &= \sum_k n_{ik} \\ p(D|\alpha) &= \prod_i p(\boldsymbol{x_i}|\alpha) \\ &= \prod_i (\frac{\Gamma(\sum_k \alpha_k)}{\Gamma(n_i + \sum_k \alpha_k)} \prod_k \frac{\Gamma(n_{ik} + \alpha_k)}{\Gamma(\alpha_k)} \end{aligned}$$

It can be shown that one Newton step is defined as:

$$\boldsymbol{\alpha^{new}} = \boldsymbol{\alpha^{old}} - \boldsymbol{H}^{-1}\boldsymbol{g} \tag{1}$$

where $\boldsymbol{H}^{-1}$ is the inverted Hessian matrix and $\boldsymbol{g}$ is the gradient of the log-likelihood function:

$$g_k = \frac{d \log p(D|\boldsymbol{\alpha})}{d\alpha_k} \tag{2}$$

$$= \sum_i \Psi(\sum_k \alpha_k) - \Psi(\sum_k n_{ik} + \sum_k \alpha_k) + \Psi(n_i k + \alpha_k) - \Psi(\alpha_k) \tag{3}$$

$$\text{where, } \Psi(y) = \frac{d \log \Gamma(y)}{dy} \tag{4}$$

Termination of the algorithm is ensured by setting a lower bound δ on the difference of the log-likelihood functions with new and old $\boldsymbol{\alpha}$ parameter vectors:

$$\log p(D|\boldsymbol{\alpha^{new}}) - \log p(D|\boldsymbol{\alpha^{old}}) \geq \delta$$

We initialize the algorithm with the method of moments estimator of $\boldsymbol{p}$. In some cases, the first Newton step might create non-admissible $\boldsymbol{\alpha}$ parameter vector where $\alpha_k < 0$ for some k. In such case, we restart the Newton-Rhapson and choose the lowest base call frequency observed for each base as the new starting values as suggested in [2].

3 *in silico* Benchmark

3.1 Simulation of *in silico* data

In the following, we will provide additional technical details on how the benchmark data set was designed (see Figure 1). In order to enable a feasible comparison of variant callers, we define a common search region based on the generated BAM files. Apart from coverage requirements, we aim to match the sum of TP, TP, TN, and FN among all variants callers. In the gDNA vs. cDNA setup we remove all gDNA variants from the search region according to the general approach to detect RNA editing sites discussed above. Finally, we filter the set of implanted variants. When no replicate information is available, we require that at least two reads harbouring the same variant allele are present. In the other case, the variant base needs to be identified in each replicate.

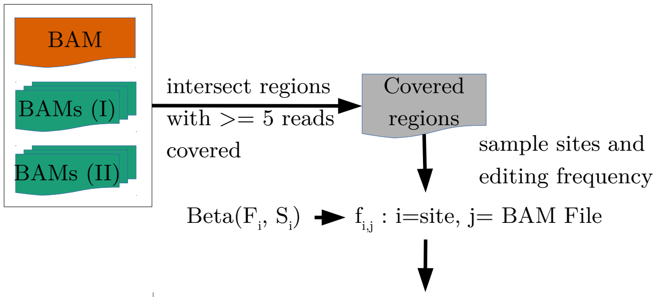

**Site**	**Target frequency**	**Shape parameter S = (α + β)**	**BAM 1 frequency**	**...**	**BAM j frequency**	**...**	**BAM 15 frequency**
**1**	F_1	S_1	$f_{1,1}$	...	$f_{1,j}$	...	$f_{1,15}$
**...**	...	...	...	...	...	...	...
**i**	F_i	S_i	$f_{i,1}$	...	$f_{i,j}$	...	$f_{i,15}$
**...**	...	...	...	...	...	...	...
**30000**	F_{30000}	S_{30000}	$f_{30000,1}$	...	$f_{30000,j}$	...	$f_{30000,15}$

Figure 1: Detailed description of data setup for the *in silico* benchmark. Generation of base change frequencies to create SNPs and SNVs in cDNA samples. Exemplified is the generation of SNP sites for one cDNA sample and 15 BAM files. Candidate regions with at least 5 reads covered from all 31 BAM files (1 gDNA + 3x5 cDNA I + 3x5 cDNA II) are extracted. 30,000 polymorphic and 30,000 non-overlapping variants site are sampled from candidate regions. Base change frequencies are sampled from a Beta distribution $\mathcal{B}(\alpha, \beta)$. For each site i a target frequency $F_i :\in I$ from an interval I and a shape parameter $S : \{10, 50, 100\}$ is sampled. With $\overline{F_i} = 1 - F_i$, the Beta distribution $\mathcal{B}(F_i \cdot S, \overline{F_i} \cdot S)$ is used to create editing frequencies for each BAM file. In SNP generation all 31 BAM files have the same target frequency F_i per site i but different actual frequencies $f_{i,j}$ per BAM file. Variant generation is done likewise, with F_i^I and F_i^{II} corresponding to target frequencies of cDNA sample I and II, respectively. To ensure sufficient difference between target frequencies F_i^I and F_i^{II} sampling is performed such that $|F_i^I - F_i^{II}| > 0.1$ is achieved.

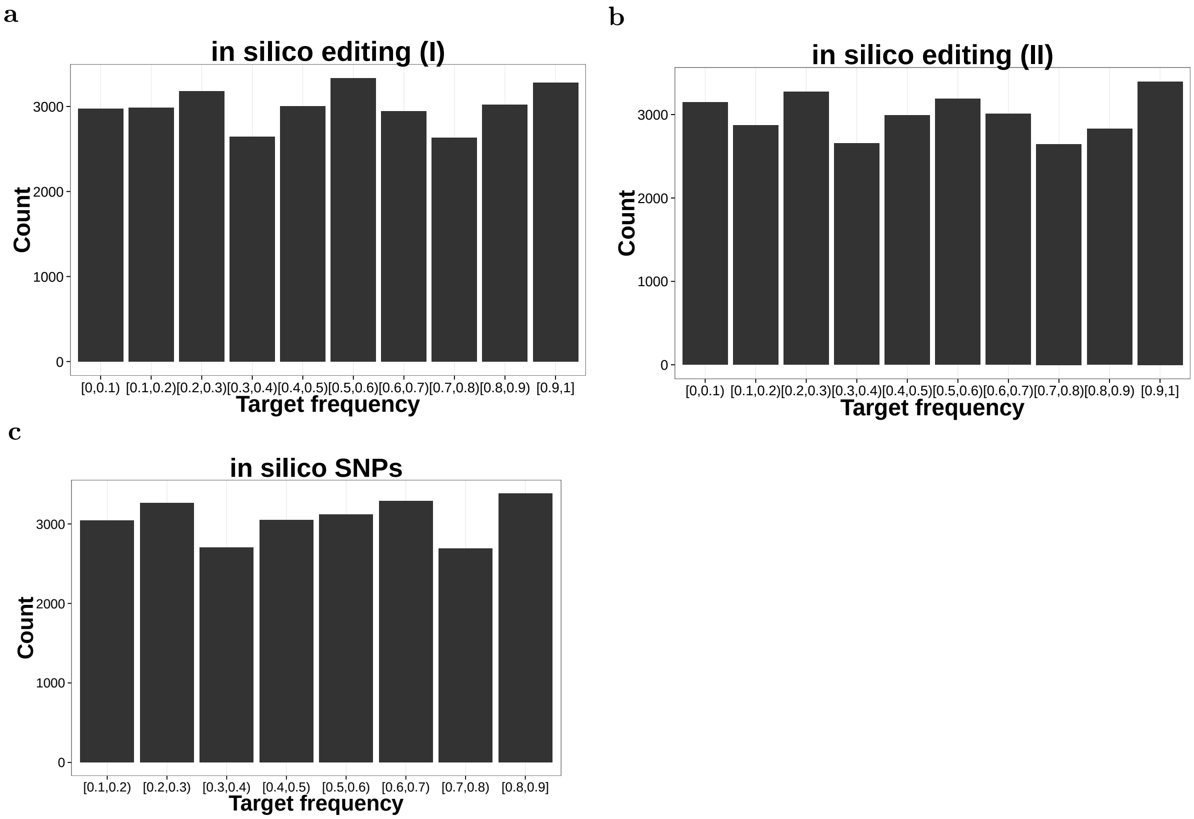

Figure 2: Distribution of target frequencies of implanted variants into RRD benchmark. Editing frequencies of variants implanted into (a) sample I and (b) sample II. (c) Allele frequencies of SNPs implanted in the cDNA vs. cDNA benchmark setup.

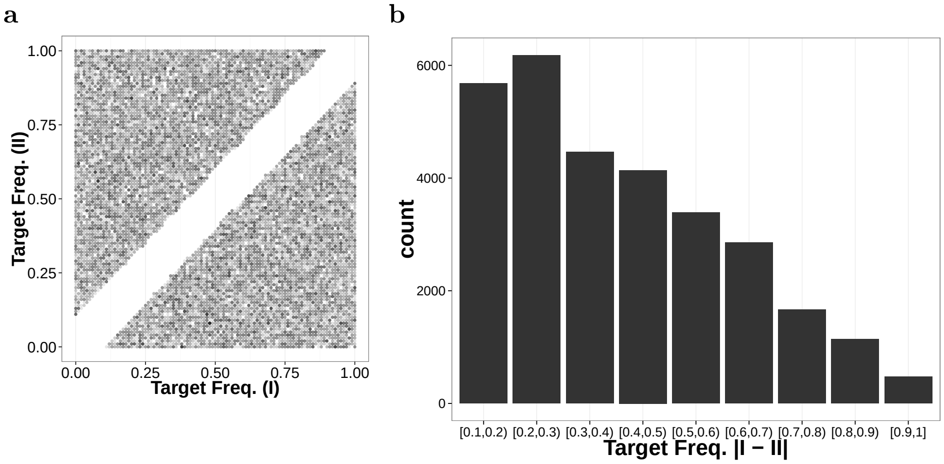

Figure 3: (a) shows a density plot of the pairwise target frequency F_i^I and F_i^{II} from sample I and II, respectively. (b) shows the distribution of absolute differences $|F_i^I - F_i^{II}|$ of target frequency from sample I and II.

Simulation of DNA-seq data

We used ART Version 2.1.8 [3] to simulate paired-end, 2x100nt gDNA reads from chromosome 1 of the human genome reference (hg19). The following parameters have been used to simulate 30x coverage:

```
art_illumina -na -i <FASTA> -p -l 100 -f 30 -m 400 -s 30
```

The DNA FASTQ-file has been mapped with bowtie2 [4] against the whole human genome reference:

```
bowtie2 --mm -p 16 --local -x <hg19-index>
```

Simulation of RNA-seq data

We used the FLUX simulator v1.2.1[5], a tool for the simulation of RNA-Seq data, to generate *in silico* reads for the human transcriptome of chromosome 1. We used the default parameters but adjusted for the read length and read number. Subsequently, RNA-seq reads have been splice-aligned with tophat2 v2.0.13 against the whole genome and transcriptome with the following parameters:

```
tophat2 -p 10 --read-realign-edit-dist 0 -z0 \
  -G Homo_sapiens.GRCh37.75.gff
```

Reads mappings to other chromosome than 1 have been removed from the final output.

3.1.1 FLUX simulator - parameter file

The FLUX simulator was used to simulate RNA-seq data sets for the in *silico* benchmarks.
The respective program parameters were taken from `http://sammeth.net/confluence/pages/viewpage.action?pageId=786691`. The read length has been adjusted to 100nt and the number of reads has been set to 15,000,000.

SAMtools/BCFtools

We employ the software package SAMtools/BCFtools v0.1.19 [6] to predict variants in RNA-DNA and RNA-RNA comparisons. The following command line arguments are executed as part of our benchmark.

```
samtools mpileup -Q 20 -q 20 -d 1000 -RDsugIBA \
  -f <FASTA> <A.bams> <B.bams> | \
  bcftools view -cevI -1 ‘echo <A.bams> | wc -w ‘ -
```

Subsequently, the results are filtered with the *varFilter.pl* script, which is included in the SAMtools/BCFtools distribution and is suggested by the online manual to perform post-hoc filtering:

```
bcftools/vcfutils.pl varFilter -1 0 -4 0.05 -e 0
```

Following the recommendation in [7], we changed the default value for the end distance bias filter to "-4 0.05". Finally, we employ a custom AWK script to extract the LRT1 and LRT2 test statistic from the filtered VCF file. The LRT1,2 test predicts if two groups are significantly different by comparing their allele frequencies (see [6] for details). We used the LRT1 test-statistic throughout the manuscript but provide the LRT2 results in section 3.4. The subsequent tools REDItools and MuTect are only applicable in an RDD scenario.

REDItools

We use REDItools-1.0.3 [8] with the following parameters:

```
python REDItoolDenovo.py -o <OUTPUT_DIR> -i <B.bam> -f <FASTA> \
  -t 1 -c 5 -q 20 -e -d -T 6-6 -W -E -r 4
```

REDItoolDenovo.py does not use gDNA sequencing data to predict RDDs by assuming that putative RNA editing sites are not polymorphic. For our benchmark this is a valid assumption because we only implant variants into cDNA samples. Because REDItools only utilizes RNA-seq data, all predictions should be filtered against known genomic variant sites in real-world examples. This information is typically available through dbSNP [9].

MuTect

MuTect [10] is a popular somatic variant caller. We employ muTect-1.1.4 to predict variants with the following parameters:

```
java -Djava.io.tmpdir=~/tmp -jar muTect-1.1.4.jar \
  --analysis_type MuTect -nt 1 --enable_extended_output \
  --reference_sequence <FASTA> --input_file:normal <A.bam> \
  --input_file:tumor <B.bam> -U ALLOW_SEQ_DICT_INCOMPATIBILITY \
  -out <OUTPUT>
```

We supply the cDNA BAM file, which contains the variants, as the tumor input file and the gDNA BAM files as reference condition.

JACUSA

We call our own software solution with the following parameters:

```
java -jar JACUSA.jar call-2 -w 10000 -W 1000000 -c 5 -m 20 -d 1000 \
  -q 20 -r <OUTPUT> -p 1 -T 0 -a D <A.bams> <b.bams>
```

3.2 Benchmark evaluation

We use the ROCR R package [11] to evaluate our benchmark results. ROCR computes, among others, the following relevant performance measures:

$$\text{True positive rate (TPR)} = \frac{TP}{TP + FN}$$

$$\text{False positive rate (FPR)} = \frac{FP}{TN + FP}$$

$$\text{Accuracy} = \frac{TP + FP}{TP + FP + FN + TN}$$

$$\text{Precision} = \frac{TP}{TP + FP}$$

$$\text{F-score} = 2 \cdot \frac{precision \cdot TPR}{precision + TPR}$$

where TP, TN, FP, and FN represent the number of true positives, true negatives, false positives and false negatives, respectively.

3.3 Additional results for gDNA vs. cDNA comparison

This section contains additional results for the benchmark represented by Figure 3 in the main text.

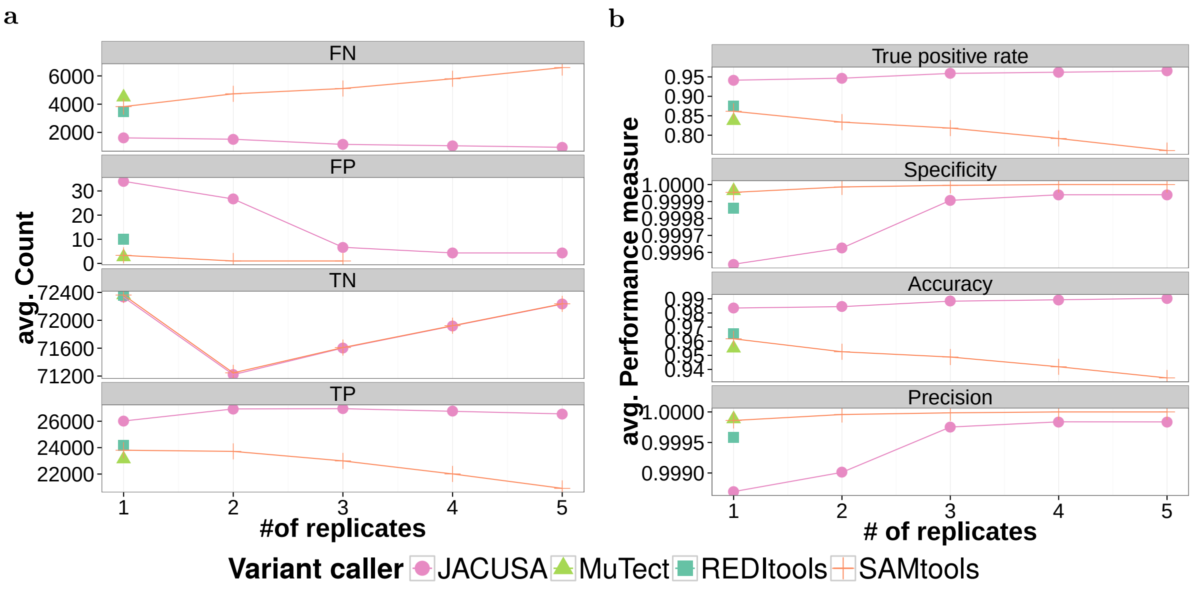

Figure 4: Performance results for gDNA vs. cDNA comparisons. (a) count results and (b) performance measure. (TP = true positives, FP = false positives, TN = true negatives, FN = false negatives)

Table 1: Detailed performance results for gDNA and cDNA comparision. Results are sorted by accuracy for each block of replicates.

# of replicates	Variant caller	TP	TN	FP	FN	TPR	Precision	Accuracy
1	JACUSA	26,016	72,331	34	1,616	0.9415	0.9987	0.9835
1	REDItools	24,165	72,355	10	3,466	0.8745	0.9996	0.9652
1	SAMtools	23,805	72,362	3	3,826	0.8615	0.9999	0.9617
1	MuTect	23,137	72,362	2	4,495	0.8373	0.9999	0.9550
2	JACUSA	26,929	71,220	26	1,519	0.9466	0.9990	0.9845
2	SAMtools	23,716	71,245	1	4,732	0.8337	1.0000	0.9525
3	JACUSA	26,949	71,602	6	1,154	0.9589	0.9998	0.9884
3	SAMtools	22,996	71,608	0	5,107	0.8183	1.0000	0.9488
4	JACUSA	26,750	71,918	4	1,057	0.9620	0.9998	0.9894
4	SAMtools	22,010	71,922	0	5,797	0.7915	1.0000	0.9419
5	JACUSA	26,555	72,231	4	949	0.9655	0.9998	0.9904
5	SAMtools	20,917	72,235	0	6,587	0.7605	1.0000	0.9340

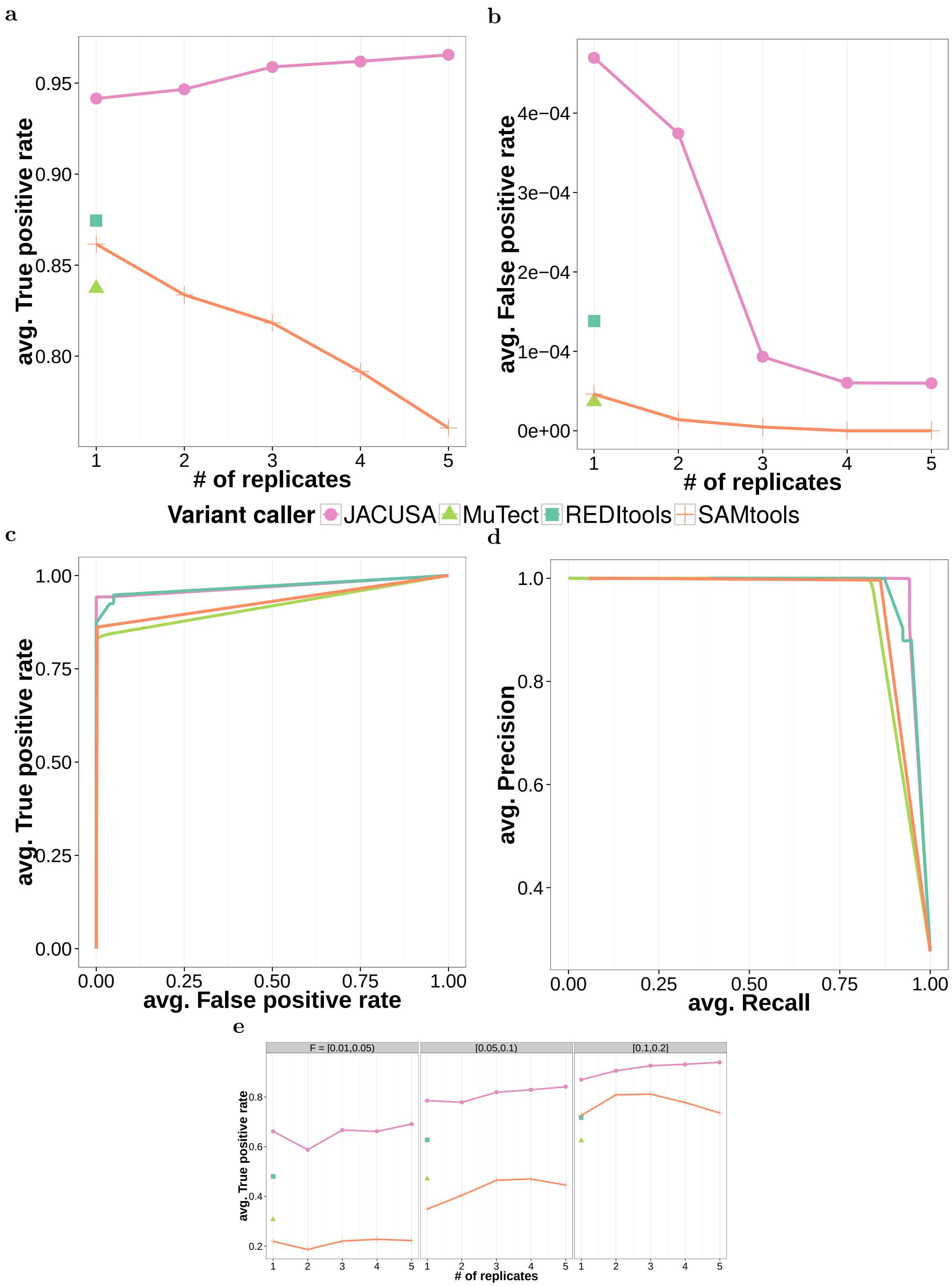

Figure 5: Performance results for gDNA vs. cDNA comparisons with differing number of replicates. (a) True positive rate and (b) False positive rate for increasing number of replicates. (c) True positive and False positive rate and (d) precision and recall for 1 replicate. (e) True positive rate stratified by variant frequency.

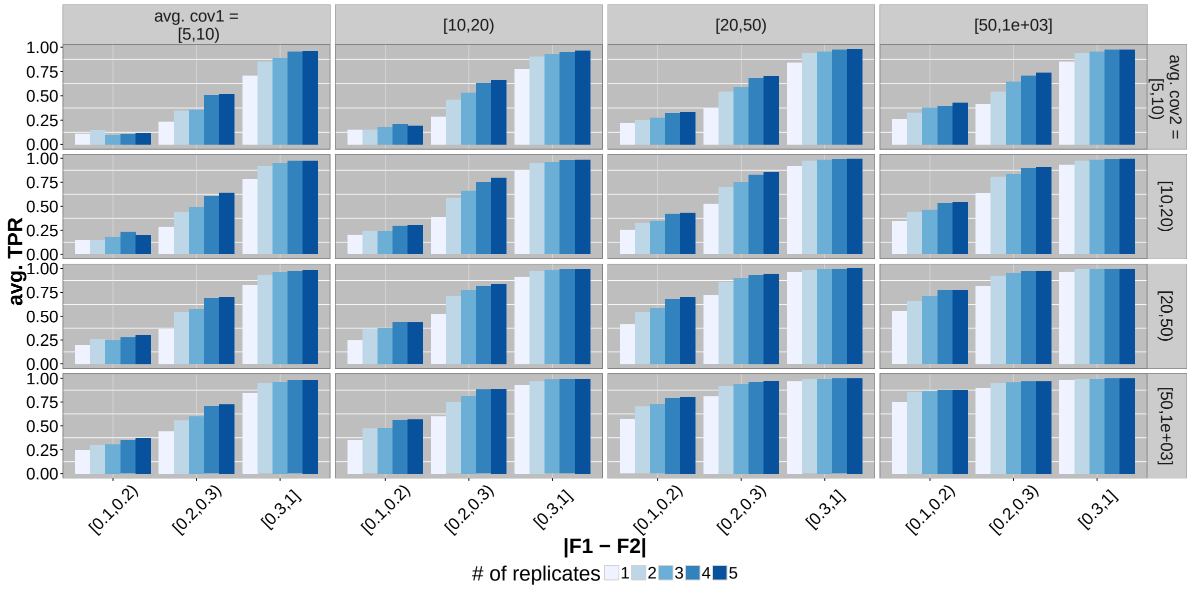

Figure 6: Depicted is the impact of average read coverage and the number of replicates on the true positive rate (TPR) of JACUSA predicted RRDs in the benchmark. TRP is positively correlated with the number of replicates and the average read coverage. Increasing the average read coverage has a stronger positive effect on the TPR than increasing the number of replicates — compare TPR along the diagonal plots vs. within plots. Sites that have low average read coverage (5-10 reads) show almost no change in TPR when the number of replicates is increased.

3.4 Additional results for cDNA vs. cDNA comparison

This section contains additional results for the benchmark represented by Figure 4 in the main text.

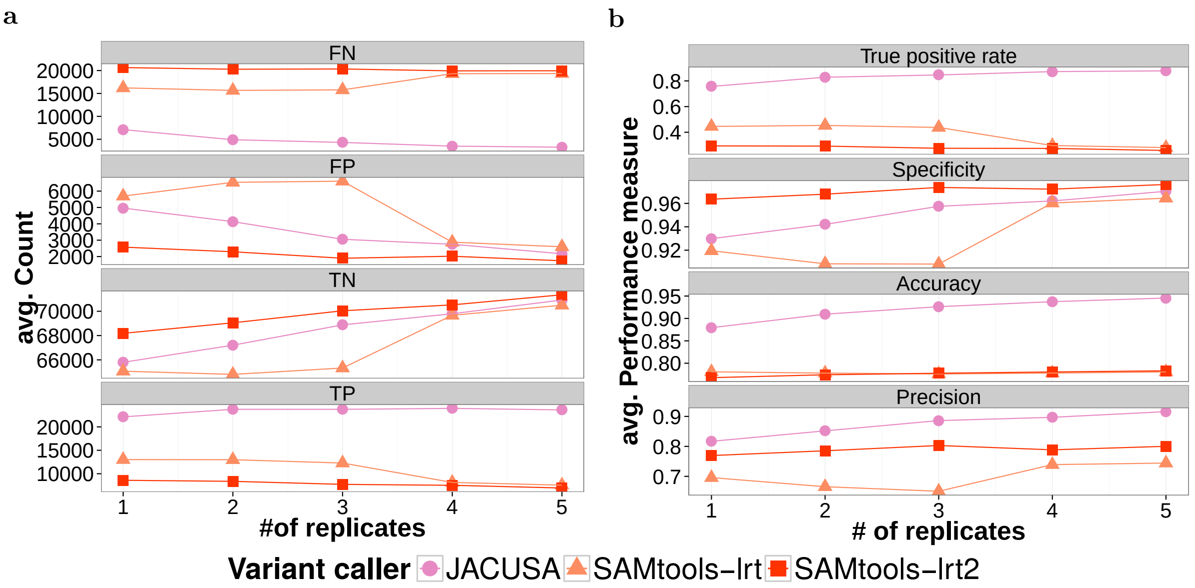

Figure 7: Performance results for cDNA vs. cDNA comparisons. (a) count results and (b) performance. (TP = true positives, FP = false positives, TN = true negatives, FN = false negatives)

Table 2: Detailed performance results for cDNA and cDNA comparisions. Results are sorted by accuracy for each block of replicates.

# of replicates	Variant caller	TP	TN	FP	FN	TPR	Precision	Accuracy
1	JACUSA	22,134	65,797	4,962	7,105	0.7570	0.8169	0.8793
1	SAMtools-lrt	13,022	65,066	5,693	16,217	0.4454	0.6958	0.7809
1	SAMtools-lrt2	8,597	68,183	2,576	20,642	0.2940	0.7694	0.7678
2	JACUSA	23,750	67,206	4,133	4,910	0.8287	0.8518	0.9096
2	SAMtools-lrt	12,994	64,802	6,537	15,666	0.4534	0.6655	0.7780
2	SAMtools-lrt2	8,378	69,047	2,292	20,282	0.2923	0.7853	0.7743
3	JACUSA	23,748	68,879	3,056	4,316	0.8462	0.8860	0.9263
3	SAMtools-lrt2	7,729	70,038	1,897	20,335	0.2754	0.8030	0.7777
3	SAMtools-lrt	12,281	65,328	6,607	15,782	0.4376	0.6503	0.7761
4	JACUSA	23,948	69,795	2,750	3,506	0.8723	0.8970	0.9374
4	SAMtools-lrt2	7,526	70,525	2,019	19,928	0.2741	0.7885	0.7805
4	SAMtools-lrt	8,143	69,667	2,878	19,311	0.2966	0.7392	0.7781
5	JACUSA	23,627	70,927	2,169	3,276	0.8782	0.9159	0.9455
5	SAMtools-lrt2	6,953	71,356	1,740	19,950	0.2584	0.8002	0.7831
5	SAMtools-lrt	7,560	70,499	2,597	19,342	0.2810	0.7443	0.7806

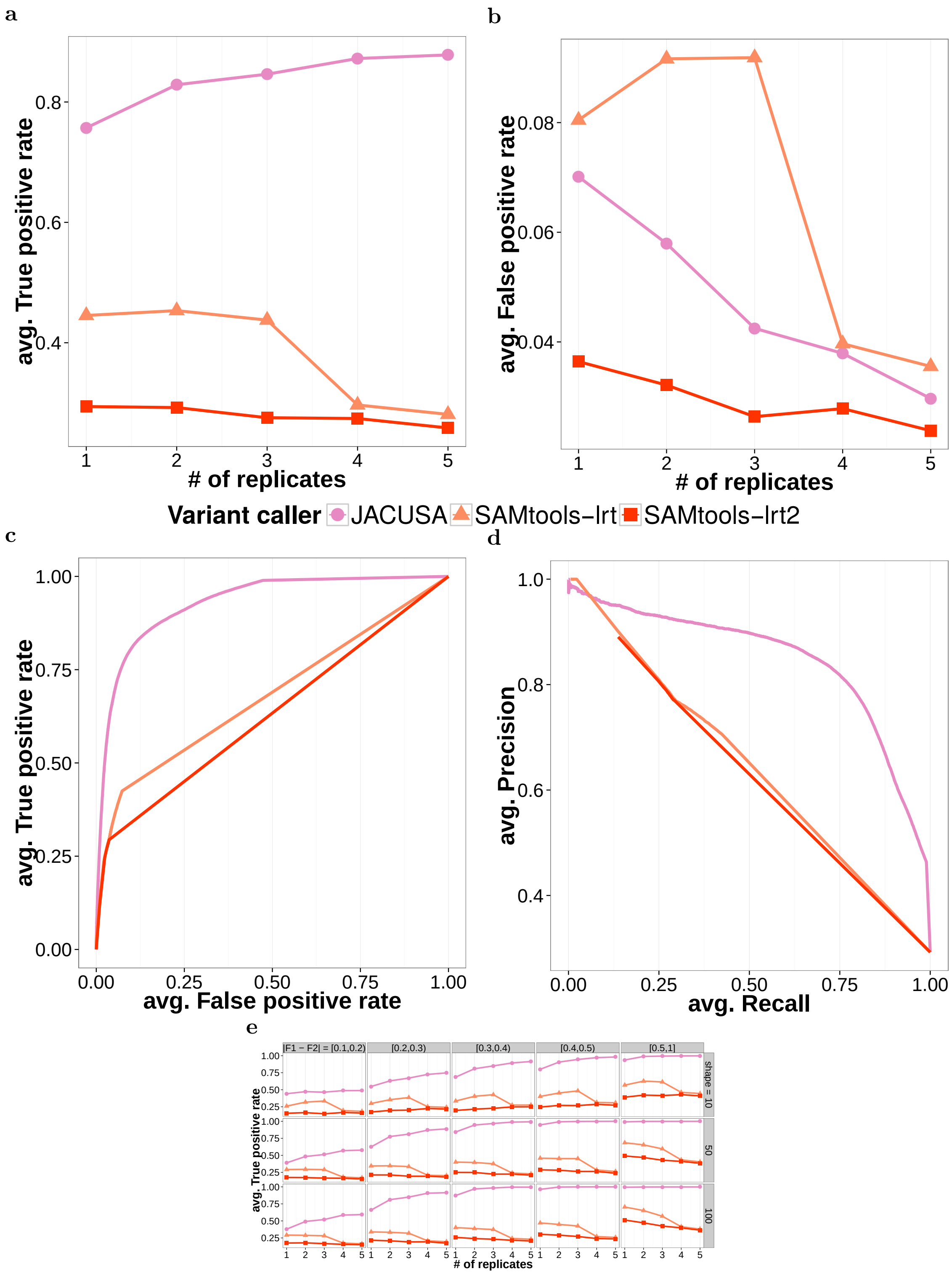

Figure 8: Performance results for cDNA vs. cDNA comparisons with differing number of replicates. (a) True positive rate and (b) False positive rate for increasing number of replicates. (c) True positive and False positive rate and (d) precision and recall for 1 replicate. (e) True positive rate stratified by the target frequency difference and shape parameter of the Beta-distribution.

3.5 Derived thresholds from *in silico* benchmark

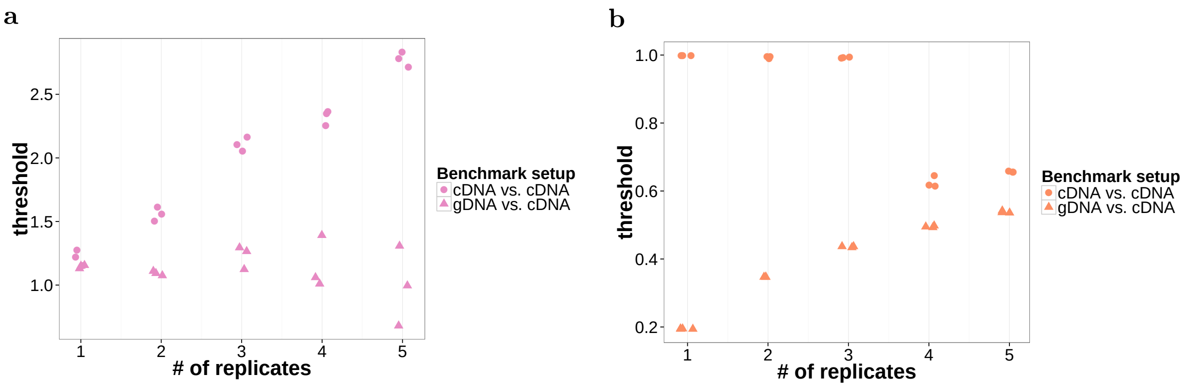

Figure 9: Optimal JACUSA (a) and SAMtools/BCFtools (b) score thresholds for different number of replicates and benchmark types. We optimized the score threshold by maximizing the benchmark accuracy. Each point represents one variant caller run with the given number of replicates and the corresponding benchmark setup. We defined the thresholds based on the mean of each combination of benchmark setup and number of replicates (for HEK-293 data with 2 replicates: gDNA vs. cDNA = 1.15 and cDNA vs. cDNA = 1.56).

Table 3: Optimal thresholds for REDItools and MuTect for gDNA vs. cDNA comparisons when no replicates are available. Last column shows the average threshold.

Variant caller	Thresholds (3 runs)	Average
MuTect	6.300519 ; 6.300457 ; 6.300536	6.30
REDITools	0.311362 ; 0.311304 ; 0.311427	0.31

3.6 Running time analysis

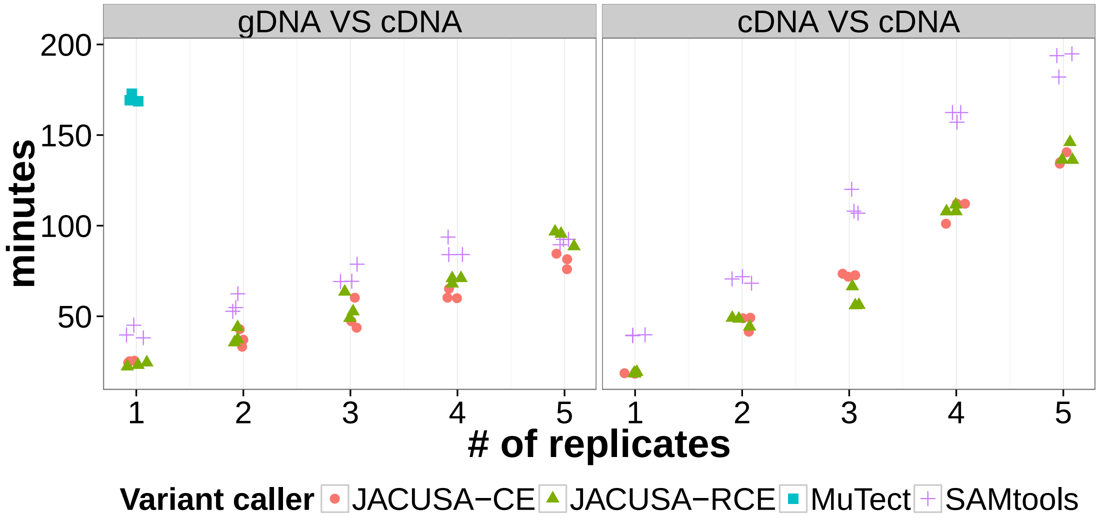

Figure 10: Running time for tested variant caller depending on benchmark type and number of replicates. REDItools are not shown because they perform an order of magnitude worse (3,103, 3,064, and 3,020 [minutes]).

4 Analysis of HEK cell line

4.1 Sequencing statistics

Table 4: Read statistic for HEK-293 sequence data. Data has been deposited under SRP050149. $cDNA\_i$: $i \in \{1, 2\}$ indicates technical replicates. Biological replicates are distinguished by the suffix e.g.: siADAR-j ($j \in \{1, 2\}$) of the Name column in the table.

Name	Library	Raw reads	Mapped reads	Uniquely mapped reads
gDNA_1	gDNA	235,832,142	206,323,227 (87%)	176,996,398 (75%)
gDNA_2 (paired)	gDNA	467,110,994	432,618,527 (93%)	382,748,613 (82%)
gDNA_3 (paired)	gDNA	465,522,096	430,904,551 (93%)	381,544,943 (82%)
cDNA_1 siADAR-1	cDNA	20,676,171	19,854,820 (96%)	14,359,177 (69%)
cDNA_1 siADAR-2	cDNA	22,791,324	22,281,537 (98%)	17,409,451 (76%)
cDNA_1 siApo-1	cDNA	187,41,904	17,163,038 (92%)	12,232,714 (65%)
cDNA_1 siApo-2	cDNA	21,097,669	20,572,103 (98%)	16,220,403 (77%)
cDNA_1 untr-1	cDNA	23,573,782	23,073,493 (98%)	16,982,923 (72%)
cDNA_1 untr-2	cDNA	19,930,282	19,317,808 (97%)	15,629,933 (78%)
cDNA_2 siADAR-1	cDNA	28,415,635	27,638,365 (97%)	19,939,442 (70%)
cDNA_2 siADAR-2	cDNA	40,123,055	39,206,824 (98%)	30,546,657 (76%)
cDNA_2 siApo-1	cDNA	25,012,075	23,789,799 (95%)	16,857,041 (67%)
cDNA_2 siApo-2	cDNA	21,512,666	20986478 (98%)	16,497,346 (77%)
cDNA_2 untr-1	cDNA	30,348,463	29,699,687 (98%)	21,759,145 (72%)
cDNA_2 untr-2	cDNA	28,432,782	27,700,311 (97%)	22,311,131 (78%)

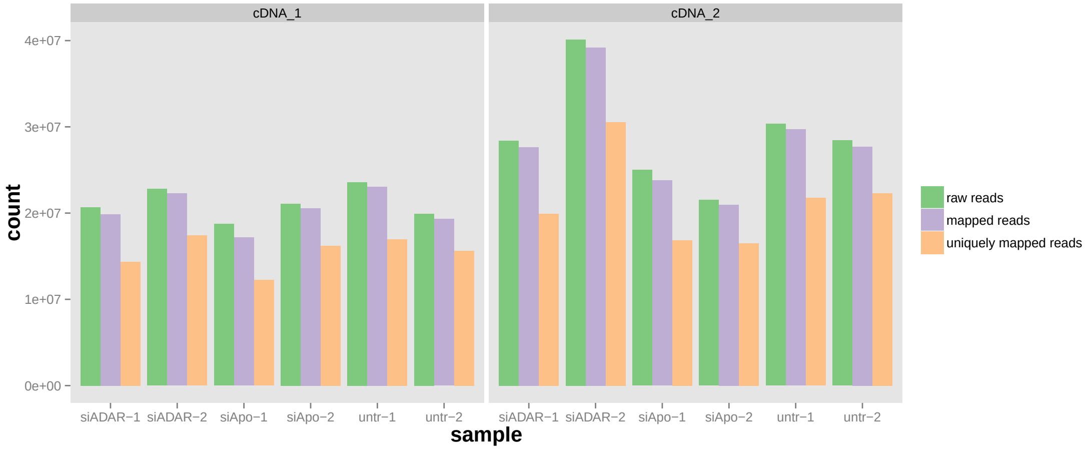

Figure 11: Read counts for sequenced RNA samples. cDNA_i : $i \in \{1, 2\}$ indicates biological replicates

4.2 Knockdown statistics

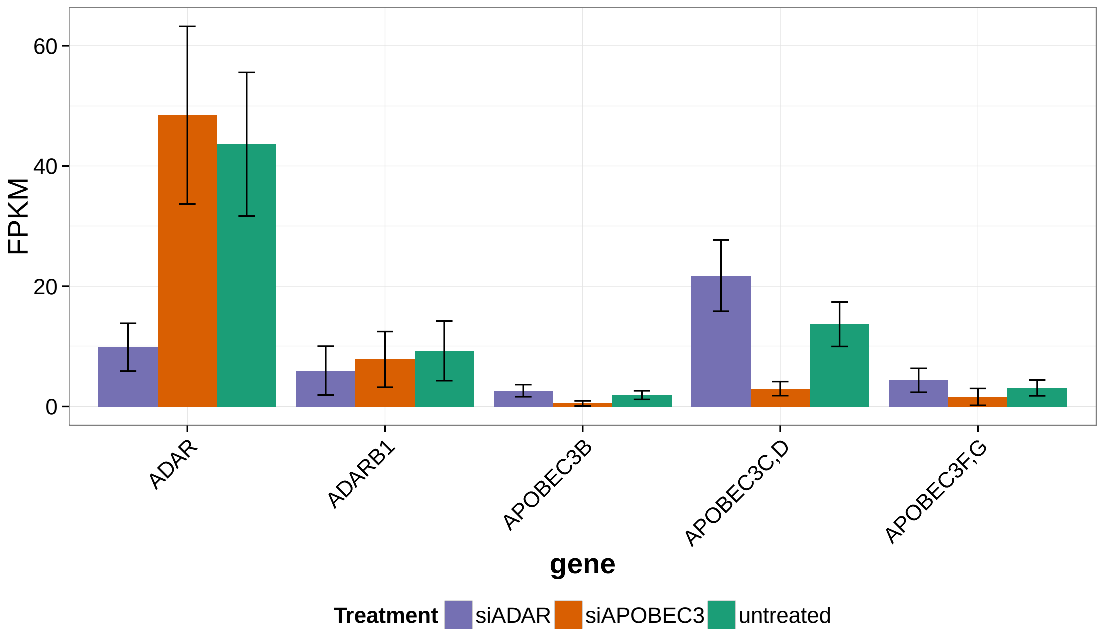

Figure 12: FPKM values of genes under study for each treatment in HEK-293 cells. (FPKM values were calculated with cufflinks. Error bars represent lower and upper bound of the 95% confidence interval of the abundance.)

4.3 Optimization of TopHat2 mismatch parameters for RNA-seq mapping

In order to study the impact of mismatches on the number of discovered editing sites we used JACUSA to identify RDDs on sets of reads with increasing number of allowed mismatches (1-10). We used the fraction of identified $A \rightarrow G$ sites as a gold standard and identified 5 as an adequate value for the number of allowed mismatches (see Figure 13).

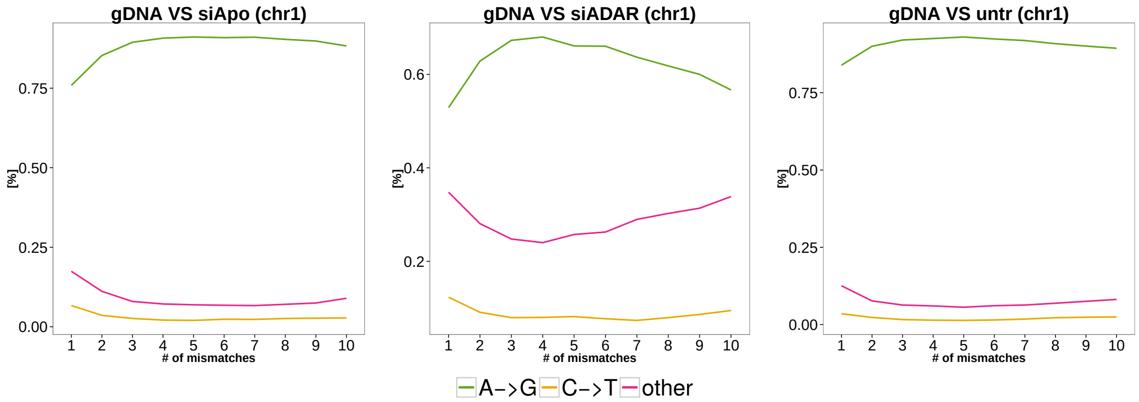

Figure 13: Depicted is the fraction of base changes dependent on the number of allowed mismatches on chromosome 1. We identified 5 mismatches as the optimal value to maximize the fraction of $A \rightarrow G$ sites among all treatments.

4.4 Marking PCR duplicates

We used MarkDuplicates from the picard tools (v1.105) to mark PCR-duplicates in gDNA and cDNA BAM files with the default parameter settings. Reads marked as PCR-duplicates are filtered by JACUSA (see 4.5).

4.5 JACUSA command line options and post-processing

We used the following command line options to identify RDDs with JACUSA in our HEK-293 samples:

```
java -jar JACUSA.jar call-2 \
-s -c 2 -P U,S -p 10 -W 1000000 \
-u DirMult -F 1024 --filterNM_1 5 --filterNM_2 5 \
-a D,M,Y,H:1 \
-T 1.15
-r $OUTPUT $DNA $RNA
```

The following options have been used to detect RRDs:

```
java -jar JACUSA.jar call-2 \
-s -c 2 -P S,S -p 10 -W 1000000 \
-u DirMult -F 1024 --filterNM_1 5 --filterNM_2 5 \
-a D,M,Y \
-T 1.56
-r $OUTPUT $RNA1 $RNA2
```

In brief, we retain reads that have a mapping quality ≥ 20 and are no potential PCR-duplicates. Furthermore, we require reads to harbor at most 5 mismatches and we require variant sites to be covered by at least 2 reads.

JACUSA output is processed by a custom R package "JacusaHelper" to infer the editing and to filter out variant sites that are covered by less than 10 reads in the gDNA BAM or less than 5 reads in each of the cDNA BAM files.

All necessary details are found in the JACUSA repository `https://github.com/dieterich-lab/JACUSA`. Please consult the README file and the subfolder "manual".

4.6 Analysis of sites rejected by filters

Figure 14 provides an overview on the different filtering mechanisms and how often they become effective.

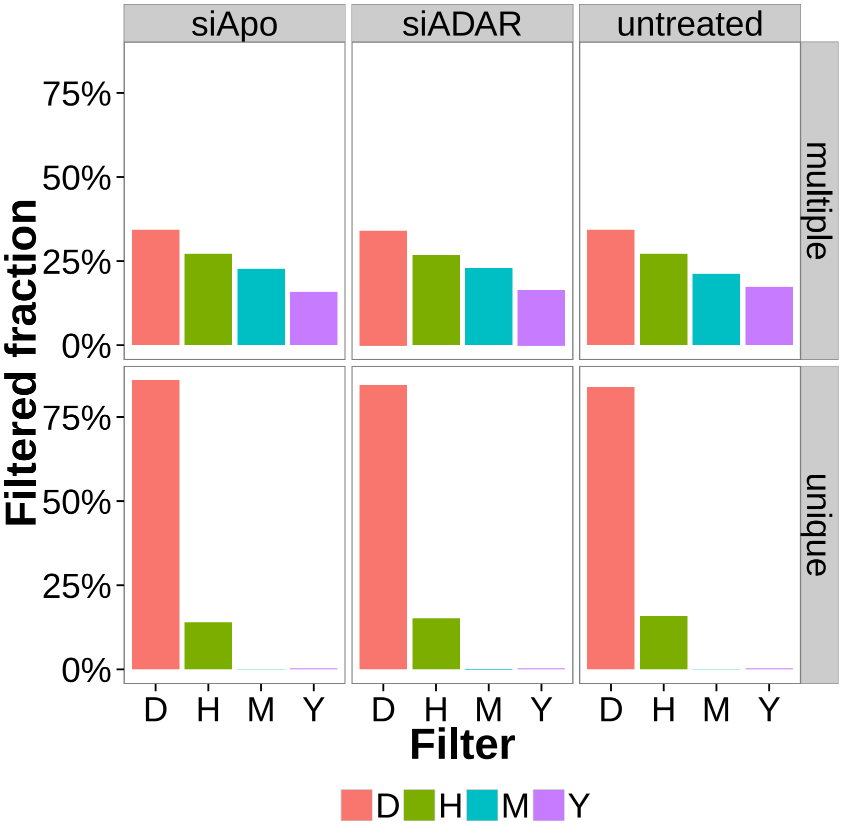

Figure 14: Shown is the distribution of excluded sites for each sample that comply with the coverage and test statistic thresholds but are rejected by some filter (see Table below). The labels ‘multiple’ and ‘unique’ indicate if a site has been excluded due to the occurrence of multiple filters (e.g.: filters B and H) or due to a single filter.

JACUSA filter	Description
B	Filters variants that are enriched at the Start/End of reads.
I	Filters variants that are in the vicinity of an INDEL.
D	This filter combines B, I, and additionally filters variants that are close to a splice site.
M	Limit the maximum allowed alleles per variant site. In a diploid cell at most two alleles are expected.
Y	Filter variant calls within homopolymers.
H	To distinguish RNA editing sites from SNPs, polymorphic read stacks in gDNA are filtered out.

4.7 Properties of variants

Figure 15 provides an overview of the identified $A \rightarrow G$ sites from all three RDD comparisons.

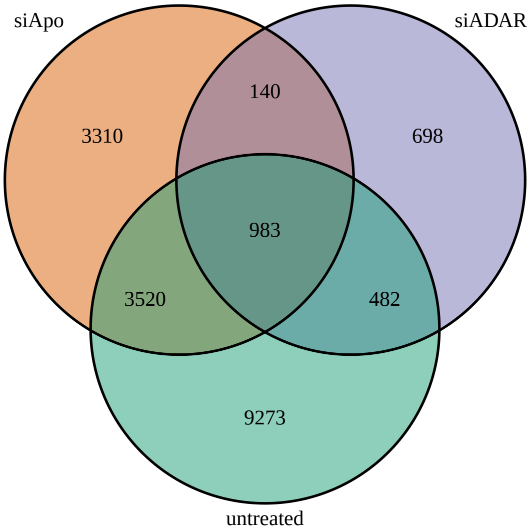

Figure 15: Overlap of $A \rightarrow G$ editing sites identified in each treatment by comparing gDNA vs. cDNA.

4.8 Comparison of RDD sites with other genomic features

RDD site overlap with genomic features

We assessed all identified variants by JACUSA by their genomic location and respective overlap with genomic features. The majority of our predicted $A \rightarrow G$ editing sites (92.9%, Fisher's Exact Test: $p < 0.0005$) overlap with Alu elements, a previously described target of RNA editing (see Figure 16a). This enrichment is not seen for other base substitutions or candidate editing events. The majority of $A \rightarrow G$ editing sites (91.3%) in the untreated sample overlaps with protein coding genes while the second highest overlapping gene type is lincRNA with 5.6% and the third frequent (3.1%) gene type is pseudogene (Figure 16b). The distribution of $A \rightarrow G$ sites is strongly affected by the experimental condition (see first row in Figure 16b). Intriguingly, the total counts for other base substitutions (e.g. $C \rightarrow T$) do not vary across different treatments (siADAR, siApo and untreated; Figure 16b). Next, we profiled the location of editing sites within the gene body of protein coding genes. The overlap of one site with a particular category of a gene is counted only once. Most of the editing sites (41.5%) are found within intronic sequences followed by 23.1% within exons (see Figure 16c). The genetic variant annotation tool snpEff revealed that only 100 $A \rightarrow G$ sites ($< 1\%$) were located within coding sequences of which 72 sites were missense variants potentially affecting the amino acid sequence of the respective protein product. We observed an increase of editing towards the 3' end (20.1% of edited sites) of a protein coding gene while $< 1\%$ of the editing sites are located in the 5'-untranslated region (5-UTR). In order to account for bias in annotating 5'- and 3'- UTRs, we extracted 5kb up- and downstream of protein coding genes and the enrichment towards the 3' end was persistent with 3.0% for upstream and $11,7\%$ for downstream editing sites.

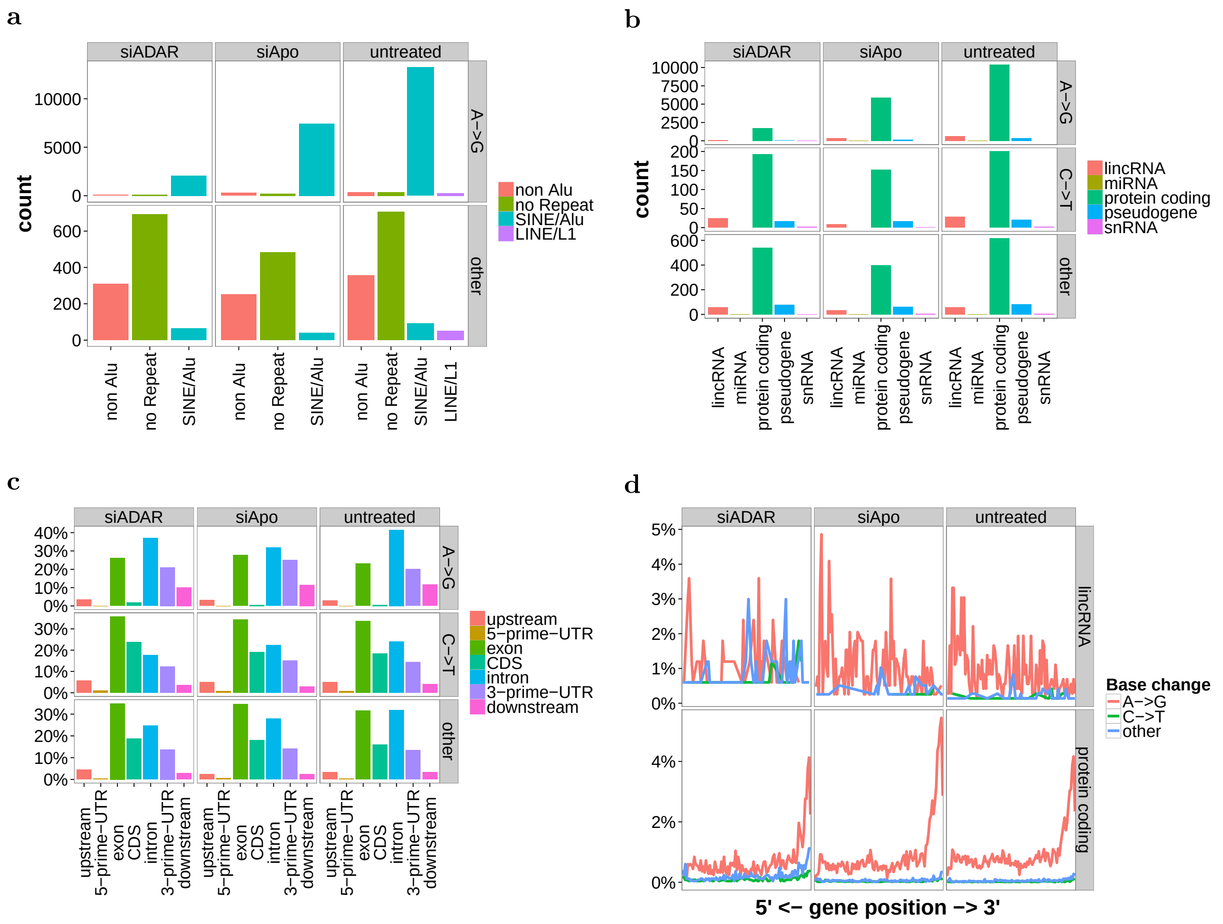

Figure 16: (a) Overlap of variants ($A \rightarrow G$ or other base changes) detected in RDD comparisons that overlap Alus or other repeats. (b) Distribution of variants overlapping gene types. (c) Distribution of variants along specific parts of protein coding genes. (d) Editing profile along protein coding genes and lincRNA.

Finally, we compared the editing profiles of protein coding genes and lincRNA based on the distribution of editing sites along length normalized genes (see Figure 16d). The enrichment of editing towards the 3' end is distinct to $A \rightarrow G$ modification and protein coding genes (n = 11,208 editing events). The editing profile for lincRNA appears to have an opposite distribution with editing enriched towards the 5' end (n = 723 editing events).

a)

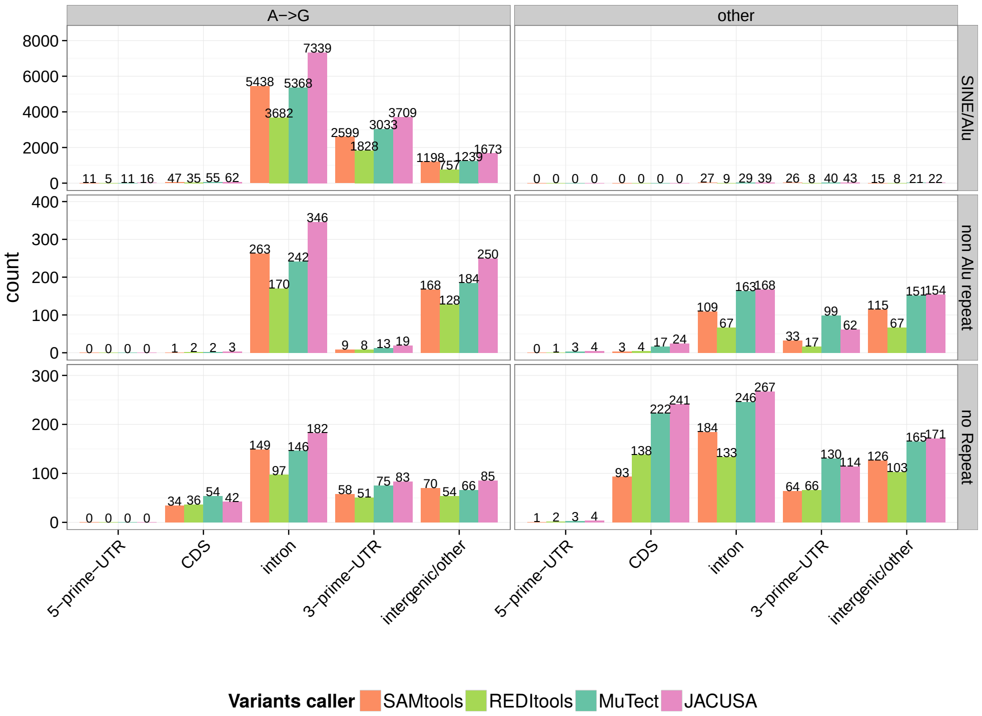

b)

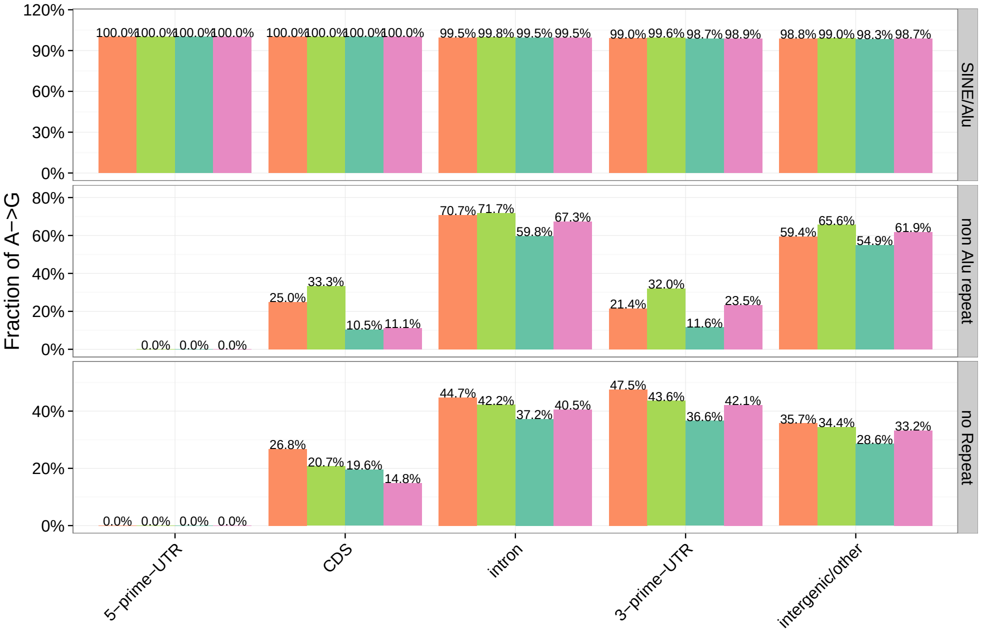

Figure 17: Detailed description of genetic and repeat annotation of RDDs that have been identified in untreated HEK-293 cells. Sites that overlap more than one repeat are discarded. a) compares the absolute counts for $A \rightarrow G$ and other variants. b) shows the fraction of $A \rightarrow G$ sites.

Table 5: Details for RDD sites identified by SAMtools/BCFtools in untreated HEK-293 cells.

Variants in:	Alu repeat		non Alu repeat		no repeat	
	Total	$A \rightarrow G$	Total	$A \rightarrow G$	Total	$A \rightarrow G$
Upstream	420	415 (98.8%)	8	4 (50%)	23	4 (17.4%)
Protein Coding						
5-'UTR	11	11 (100%)	0	0	1	0
CDS	47	47 (100%)	4	1 (25%)	127	34 (26.8%)
Intron	5,465	5,438 (99.5%)	372	263 (70.7%)	333	149 (44.7%)
3'-UTR	2,625	2,599 (99%)	42	9 (21.4%)	122	58 (47.5%)
Downstream	1,608	1,601 (99.6%)	40	33 (82.5%)	42	24 (57.1%)
Intergenic/other	1,213	1,198 (98.8%)	283	168 (59.4%)	196	70 (35.7%)
Total	11,389	11,309 (99.3%)	749	478 (63.8%)	844	339 (40.2%)
Unique	9,744	9,684 (99.4%)	734	475 (64.7%)	713	286 (40.1%)

Table 6: Details for RDD sites identified by MuTect in untreated HEK-293 cells.

Variants in:	Alu repeat		non Alu repeat		no repeat	
	Total	$A \rightarrow G$	Total	$A \rightarrow G$	Total	$A \rightarrow G$
Upstream	281	281 (100%)	3	1 (33.3%)	19	1 (5.26%)
Protein Coding						
5-'UTR	5	5 (100%)	1	0	2	0
CDS	35	35 (100%)	6	2 (33.3%)	174	36 (20.7%)
Intron	3,691	3,682 (99.8%)	237	170 (71.7%)	230	97 (42.2%)
3'-UTR	1,836	1,828 (99.6%)	25	8 (32%)	117	51 (43.6%)
Downstream	1,060	1,059 (99.9%)	26	20 (76.9%)	25	15 (60%)
Intergenic/other	765	757 (99%)	195	128 (65.6%)	157	54 (34.4%)
Total	7,673	7,647 (99.7%)	493	329 (66.7%)	724	254 (35.1%)
Unique	6,518	6,497 (99.7%)	483	324 (67.1%)	604	215 (35.6%)

Table 7: Details for RDD sites identified by REDItools in untreated HEK-293 cells.

Variants in:	Alu repeat		non Alu repeat		no repeat	
	Total	$A \to G$	Total	$A \to G$	Total	$A \to G$
Upstream	454	446 (98.2%)	24	4 (16.7%)	52	7 (13.5%)
Protein Coding						
5-'UTR	11	11 (100%)	3	0	3	0
CDS	55	55 (100%)	19	2 (10.5%)	276	54 (19.6%)
Intron	5,397	5,368 (99.5%)	405	242 (59.8%)	392	146 (37.2%)
3'-UTR	3,073	3,033 (98.7%)	112	13 (11.6%)	205	75 (36.6%)
Downstream	1,679	1,669 (99.4%)	47	32 (68.1%)	48	24 (50%)
Intergenic/other	1,260	1,239 (98.3%)	335	184 (54.9%)	231	66 (28.6%)
Total	11,929	11,821 (99.1%)	945	477 (50.5%)	1,207	372 (30.8%)
Unique	10,031	9,947 (99.2%)	878	466 (53.1%)	991	304 (30.7%)

Table 8: Details for RDD sites identified by JACUSA in untreated HEK-293 cells.

Variants in:	Alu repeat		non Alu repeat		no repeat	
	Total	$A \to G$	Total	$A \to G$	Total	$A \to G$
Upstream	558	555 (99.5%)	16	6 (37.5%)	54	7 (13%)
Protein Coding						
5-'UTR	16	16 (100%)	4	0	4	0
CDS	62	62 (100%)	27	3 (11.1%)	283	42 (14.8%)
Intron	7,378	7,339 (99.5%)	514	346 (67.3%)	449	182 (40.5%)
3'-UTR	3,752	3,709 (98.9%)	81	19 (23.5%)	197	83 (42.1%)
Downstream	2,162	2,152 (99.5%)	54	40 (74.1%)	63	31 (49.2%)
Intergenic/other	1,695	1,673 (98.7%)	404	250 (61.9%)	256	85 (33.2%)
Total	15,623	15,506 (99.3%)	1,100	664 (60.4%)	1,306	430 (32.9%)
Unique	13,336	13,245 (99.3%)	1,054	649 (61.6%)	1,071	364 (34%)

Table 9: Details for RRDs identified by SAMtools/BCFtools in siADAR vs. siAPOBEC3 treated HEK-293 cells.

Variants in:	Alu repeat		non Alu repeat		no repeat	
	Total	$A \to G$	Total	$A \to G$	Total	$A \to G$
Upstream	199	190 (95.5%)	5	0	73	2 (2.74%)
Protein Coding						
5-'UTR	6	6 (100%)	3	0	28	0
CDS	20	17 (85%)	12	1 (8.33%)	428	12 (2.8%)
Intron	1,795	1,695 (94.4%)	332	62 (18.7%)	673	35 (5.2%)
3'-UTR	1,625	1,551 (95.4%)	83	6 (7.23%)	475	34 (7.16%)
Downstream	740	713 (96.4%)	37	17 (45.9%)	102	11 (10.8%)
Intergenic/other	347	320 (92.2%)	144	50 (34.7%)	133	20 (15%)
Total	4,732	4,492 (94.9%)	616	136 (22.1%)	1,912	114 (5.96%)
Unique	3,827	3,621 (94.6%)	581	135 (23.2%)	1,597	93 (5.82%)

Table 10: Details for RRDs identified by JACUSA in siADAR vs. siAPOBEC3 treated HEK-293 cells.

Variants in:	Alu repeat		non Alu repeat		no repeat	
	Total	$A \rightarrow G$	Total	$A \rightarrow G$	Total	$A \rightarrow G$
Upstream	218	212 (97.2%)	8	1 (12.5%)	46	2 (4.35%)
Protein Coding						
5-'UTR	8	8 (100%)	1	0	18	0
CDS	19	19 (100%)	11	0	331	22 (6.65%)
Intron	1,750	1,685 (96.3%)	140	51 (36.4%)	292	31 (10.6%)
3'-UTR	1,900	1,832 (96.4%)	43	9 (20.9%)	299	45 (15.1%)
Downstream	800	782 (97.8%)	30	19 (63.3%)	61	10 (16.4%)
Intergenic/other	324	313 (96.6%)	91	53 (58.2%)	60	16 (26.7%)
Total	5,019	4,851 (96.7%)	324	133 (41%)	1,107	126 (11.4%)
Unique	3,974	3,844 (96.7%)	298	130 (43.6%)	880	101 (11.5%)

5 RRD sites in *Drosophila melanogaster*

Table 11: Details for RRDs identified by SAMtools / BCFtools in ADAR0 vs. FM7a strains

	Variants	
	Total	$A \rightarrow G$
Protein Coding		
5-'UTR	1	0
CDS	316	264 (83.5%)
Intron	453	399 (88.1%)
3'-UTR	61	58 (95.1%)
Intergenic/other	6	3 (50%)
Total	837	724 (86.5%)
Unique	781	674 (86.3%)

Table 12: Details for RRDs identified by JACUSA in ADAR0 vs. FM7a strains

	Variants	
	Total	$A \rightarrow G$
Protein Coding		
5-'UTR	2	2 (100%)
CDS	383	336 (87.7%)
Intron	530	502 (94.7%)
3'-UTR	77	74 (96.1%)
Intergenic/other	8	6 (75%)
Total	1,000	920 (92%)
Unique	931	857 (92.1%)

References

[1] Minka, T.: Estimating a Dirichlet distribution. Technical report, MIT (2000)

[2] Ronning, G.: Maximum likelihood estimation of dirichlet distributions. Journal of statistical computation and simulation **32**(4), 215–221 (1989)

[3] Huang, W., Li, L., Myers, J.R., Marth, G.T.: ART: A next-generation sequencing read simulator. Bioinformatics **28**(4), 593–594 (2012). doi:10.1093/bioinformatics/btr708

[4] Langmead, B., Salzberg, S.L.: Fast gapped-read alignment with Bowtie 2 (2012). #14603. doi:10.1038/nmeth.1923

[5] Griebel, T., Zacher, B., Ribeca, P., Raineri, E., Lacroix, V., Guigó, R., Sammeth, M.: Modelling and simulating generic RNA-Seq experiments with the flux simulator. Nucleic acids research **40**(20), 10073–83 (2012). doi:10.1093/nar/gks666

[6] Li, H.: A statistical framework for SNP calling, mutation discovery, association mapping and population genetical parameter estimation from sequencing data. Bioinformatics (Oxford, England) **27**(21), 2987–93 (2011). doi:10.1093/bioinformatics/btr509

[7] Danecek, P., Nellaker, C., McIntyre, R.E., Buendia-Buendia, J.E., Bumpstead, S., Ponting, C.P., Flint, J., Durbin, R., Keane, T.M., Adams, D.J.: High levels of rna-editing site conservation amongst 15 laboratory mouse strains. Genome Biol **13**(4), 26 (2012)

[8] Picardi, E., Pesole, G.: REDItools: High-throughput RNA editing detection made easy. Bioinformatics **29**(14), 1813–1814 (2013). doi:10.1093/bioinformatics/btt287

[9] Sherry, S.T., Ward, M.-H., Kholodov, M., Baker, J., Phan, L., Smigielski, E.M., Sirotkin, K.: dbsnp: the ncbi database of genetic variation. Nucleic acids research **29**(1), 308–311 (2001)

[10] Cibulskis, K., Lawrence, M.S., Carter, S.L., Sivachenko, A., Jaffe, D., Sougnez, C., Gabriel, S., Meyerson, M., Lander, E.S., Getz, G.: Sensitive detection of somatic point mutations in impure and heterogeneous cancer samples. Nature biotechnology **31**(3), 213–9 (2013). doi:10.1038/nbt.2514. NIHMS150003

[11] Sing, T., Sander, O., Beerenwinkel, N., Lengauer, T.: ROCR: Visualizing classifier performance in R. Bioinformatics **21**(20), 3940–3941 (2005). doi:10.1093/bioinformatics/bti623
